# Antigen presentation and interferon signatures in B cells driven by localized ablative cancer immunotherapy correlate with extended survival

**DOI:** 10.7150/thno.65773

**Published:** 2022-01-01

**Authors:** Kaili Liu, Ashley R. Hoover, Jason R. Krawic, Christa I. DeVette, Xiao-Hong Sun, William H. Hildebrand, Mark L. Lang, Robert C. Axtell, Wei R. Chen

**Affiliations:** 1 Stephenson School of Biomedical Engineering, University of Oklahoma, Norman, OK, USA.; 2 Arthritis and Clinical Immunology Research Program, Oklahoma Medical Research Foundation, Oklahoma City, OK, USA.; 3 Department of Microbiology and Immunology, University of Oklahoma Health Sciences Center, Oklahoma City, OK, USA.

**Keywords:** Single-cell RNA sequencing, B cell activation, localized ablative immunotherapy, N-dihydrogalactochitosan, breast cancer

## Abstract

**Rationale:** B cells have emerged as key regulators in protective cancer immunity. However, the activation pathways induced in B cells during effective immunotherapy are not well understood.

**Methods:** We used a novel localized ablative immunotherapy (LAIT), combining photothermal therapy (PTT) with intra-tumor delivery of the immunostimulant N-dihydrogalactochitosan (GC), to treat mice bearing mouse mammary tumor virus-polyoma middle tumor-antigen (MMTV-PyMT). We used single-cell RNA sequencing to compare the transcriptional changes induced by PTT, GC and PTT+GC in B cells within the tumor microenvironment (TME).

**Results:** LAIT significantly increased survival in the tumor-bearing mice, compared to the treatment by PTT and GC alone. We found that PTT, GC and PTT+GC increased the proportion of tumor-infiltrating B cells and induced gene expression signatures associated with B cell activation. Both GC and PTT+GC elevated gene expression associated with antigen presentation, whereas GC elevated transcripts that regulate B cell activation and GTPase function and PTT+GC induced interferon response genes. Trajectory analysis, where B cells were organized according to pseudotime progression, revealed that both GC and PTT+GC induced the differentiation of B cells from a resting state towards an effector phenotype. The analyses confirmed upregulated interferon signatures in the differentiated tumor-infiltrating B cells following treatment by PTT+GC but not by GC. We also observed that breast cancer patients had significantly longer survival time if they had elevated expression of genes in B cells that were induced by PTT+GC therapy in the mouse tumors.

**Conclusion:** Our findings show that the combination of local ablation and local application of immunostimulant initiates the activation of interferon signatures and antigen-presentation in B cells which is associated with positive clinical outcomes for breast cancer. These findings broaden our understanding of LAIT's regulatory roles in remodeling TME and shed light on the potentials of B cell activation in clinical applications.

## Introduction

A major challenge in cancer treatment is the failure of the host to detect and destroy tumor cells 1, 2. Breast cancer is a major worldwide health burden coupled with growing prevalence of malignancy-related mortality 3, 4. Its progression involves complicated biologic processes, which are not only controlled by genetic abnormalities but also by the interactions within the tumor microenvironment (TME) including the immune cells, extracellular matrix, surrounding blood vessels and stromal cells 5. Although tumor-infiltrating lymphocytes (TILs), as an important component of immune cells in the breast cancer niche, have been widely studied 6, there is still no broadly effective therapy for breast cancer 7, 8.

A localized ablative immunotherapy (LAIT) was developed based on the strategy of combining local photothermal therapy (PTT) with intra-tumor delivery of an immunostimulant 9. Targeting primary tumors with LAIT may represent a viable solution for metastatic cancers due to its capacity to induce systemic, long-term antitumor immunity 9. The LAIT combination therapy serves two major purposes. PTT disrupts target tumor homeostasis and releases tumor antigens 10, 11 while the immunostimulant enhances pro-inflammatory responses in the presence of released tumor antigens 12-15. In preliminary clinical studies of patients with metastatic, treatment-recalcitrant breast cancer and melanoma, LAIT successfully reduced and, in many cases, eliminated the treated primary tumors and untreated metastases in the lungs 16-18. The promising outcomes observed in our clinical studies prompted us to determine the underlying mechanism by which LAIT achieves effective antitumor immunity.

T and B cells play important roles in the local environment of solid tumors. While T cells have been widely investigated and therapeutically targeted for cancer treatment 6, the functions of B cells in cancer therapy have not been sufficiently evaluated. Tumor-infiltrating B cells (TIBs) are an important component of adaptive immunity and participate in both humoral and cellular immunity with diverse functions. TIBs have been reported to play both immunopotentiating and immunosuppressing roles in breast cancer 19, 20. The subtypes and mechanisms of B cells in the TME of breast cancer and how they interact with tumor cells and other stromal cell types remain largely unknown. Therefore, a comprehensive characterization of the immunological landscape of breast cancer, including the composition and roles of B lymphocytes, is essential for understanding immune mechanisms, identifying novel biomarkers and designing therapeutic strategies.

In this study, we treated murine breast tumors with LAIT, in which PTT was achieved by laser and N-dihydrogalactochitosan (GC) was used as the immunostimulant. We investigated the responses of TIBs in PTT, GC and PTT+GC treated mouse mammary tumor virus-polyoma middle tumor-antigen (MMTV-PyMT) tumors. PTT+GC significantly extended the survival of tumor-bearing animals. Using single-cell RNA sequencing (scRNA-seq), we performed clustering analyses to identify B cell subtypes in tumors after different treatments. We also identified differential gene expression (DGE) patterns by comparing individual treatment groups with untreated controls using gene functional enrichment methods. We observed that PTT, GC and PTT+GC enhanced the proportion of TIBs and stimulated gene expression signatures related to B cell activation. Both GC and PTT+GC elevated gene expression associated with antigen presentation, whereas GC elevated transcripts that regulate B cell activation and GTPase function and PTT+GC induced interferon response genes. Trajectory inference revealed that both GC and PTT+GC induced the differentiation of B cells from a resting state toward an effector phenotype along the pseudotime progression. The analyses also confirmed upregulation of interferon signatures in tumors treated by PTT+GC but not by GC in the differentiated B cells. Moreover, we discovered that breast cancer patients had significantly extended overall survival if they had elevated expression of genes that were induced by PTT+GC therapy in the mouse tumors. Our findings show that LAIT initiates the activation of interferon signatures and antigen-presentation in B cells which is positively associated with favorable clinical outcomes for breast cancer.

## Results

### PTT+GC therapy extends the survival of mice bearing MMTV-PyMT breast tumors and increases the proportion of tumor-infiltrating B cells

**Figure 1:**
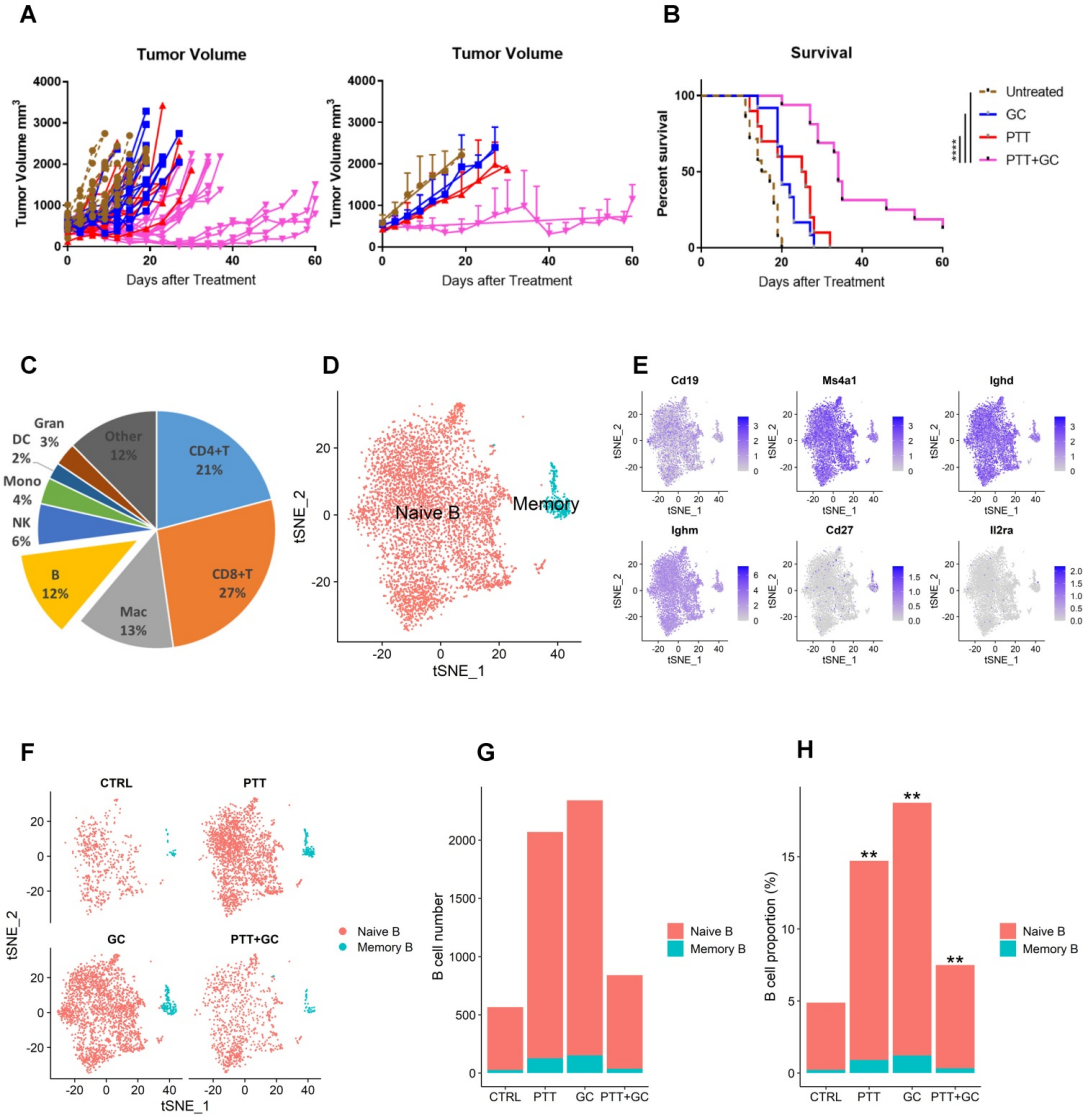
** Analysis of tumor-infiltrating B cells in different treatment groups (Control, PTT only, GC only, and PTT+GC). (A)** Individual and mean tumor size of mice following different treatments. Days after treatment up to 60 were selected for measuring the tumor volume. **(B)** Survival rates of tumor-bearing mice in different treatment groups. Log-rank (Mantel-Cox) test was used for statistical analysis. Days after treatment up to 60 were selected for measuring the mice survival. **(C)** Pie chart showing the proportions of different immune cell types in MMTV-PyMT tumors (combining all groups). B cells, consisting of 12% of total immune cells, were analyzed in this study. Mac: macrophages; NK: natural killer cells; Mono: monocytes; DC: dendritic cells; Gran: granulocytes. **(D)** Two-dimensional visualization of single-cell clusters using method of t-distributed Stochastic Neighbor Embedding (t-SNE) for B cells from all treatment groups. B cells were classified into naïve B and memory B cells. **(E)** Feature plots (t-SNE) showing expressions of major genes that identify tumor-infiltrating B cells, including *Cd19*, *Ms4a1*, *Ighd*, *Ighm*, *Cd27*, and *Il2ra*. **(F)** t-SNE plots of B cells in different treatment groups (CTRL, PTT, GC, and PTT+GC). **(G)** Number of naïve and memory B cells in each treatment group. **(H)** Proportion of naïve and memory B cells among all immune cells in each treatment group, in comparison with CTRL group (**: *p*<0.01). The number of B cells in each treatment group was divided by the number of all tumor-infiltrating CD45^+^ immune cells in the same treatment group.

The therapeutic effects of LAIT (PTT+GC in this study) on breast tumors were determined using the MMTV-PyMT transgenic tumor model. Spontaneous MMTV-PyMT tumor cells were isolated from MMTV-PyMT transgenic mice as previously described 21 and were injected into the mammary fat pad of wild type female FVB mice (Figure S1A). When tumors reached a volume of ~0.5 cm^3^, mice were separated into four treatment groups: Untreated controls (CTRL), PTT alone, GC alone, and PTT+GC. Treated animals were monitored for up to 60 days following treatment and euthanized when tumors reached ethical endpoints. PTT+GC significantly reduced tumor growth and increased the survival time compared to other groups, whereas PTT or GC alone had no statistically significant effect on changes of tumor size (Figure 1A) or survival (Figure 1B) compared with CTRL.

As shown in Figure 1A, the tumor size in the group of PTT+GC experienced statistically significant reduction when compared with CTRL at day 10. Day 10 was also a time point when untreated tumor-bearing mice in CTRL group started to die as time proceeds. The mice survival status differences among different group allowed us to infer that, at day 10, the immunologically cellular and molecular responses have affected the tumor size and animal survival. To assess changes in numbers and gene expression profiles in TIBs, we collected tumors 10 days after treatment. Cells were prepared as described in methods section and subjected to scRNA-seq analysis, following the workflow depicted in Figure S1B. After obtaining 49,380 CD45^+^ immune cells (11,584 in CTRL; 14,071 in PTT; 12,501 in GC; 11,224 in PTT+GC) and annotating these immune cell subtypes, we calculated the proportions of CD4^+^ T cells, CD8^+^ T cells, macrophages, B cells, natural killer (NK) cells, monocytes, dendritic cells, and granulocytes (Figure 1C). B cells accounted for 12% of all CD45^+^ tumor-infiltrating cells (Figure 1C). To further investigate the effect of different treatments on B cell composition and function, we conducted the clustering analysis for B cell lineage using *Seurat* R package 22. B cells were classified into two major subtypes, naïve B cell and memory B cell (Figure 1D), based on the expression of genes encoding B cell subtype markers, such as *Cd19*, *Ms4a1* (CD20), *Ighd*, *Ighm*, memory cell marker *Cd27*, and the activation cell marker *Il2ra* (CD25) (Figure 1E). Most of the cells expressed naïve B cell markers *Cd19*, *Ms4a1* (CD20), *Ighd*, and *Ighm*, with only a small number of cells expressing *Cd27* and little to no *Il2ra* expression (Figure 1E). Therefore, we obtained 5477 naïve B cells (540, 1943, 2190, and 804 for CTRL, PTT, GC, and PTT+GC, respectively) and 342 memory B cells (26, 127, 152, and 37 for CTRL, PTT, GC, and PTT+GC, respectively), as shown in Figure 1F. This result showed that the naïve B cell proportions were 4.7%, 13.8%, 17.5% and 7.2% while the memory B cell proportions were 0.2%, 0.9%, 1.2% and 0.3% for the treatments of CTRL, PTT, GC and PTT+GC, respectively (Figure 1G). Furthermore, PTT, GC and PTT+GC treatments all significantly (*p* < 0.01) increased the proportion of B cells within CD45^+^ cells compared with untreated CTRL (Figure 1H), suggesting that single and combination of treatment modality may influence the infiltrating B cell frequencies and transcriptome dynamics in TME.

### Differential gene expression and functional enrichment analysis of tumor-infiltrating B cells

**Figure 2:**
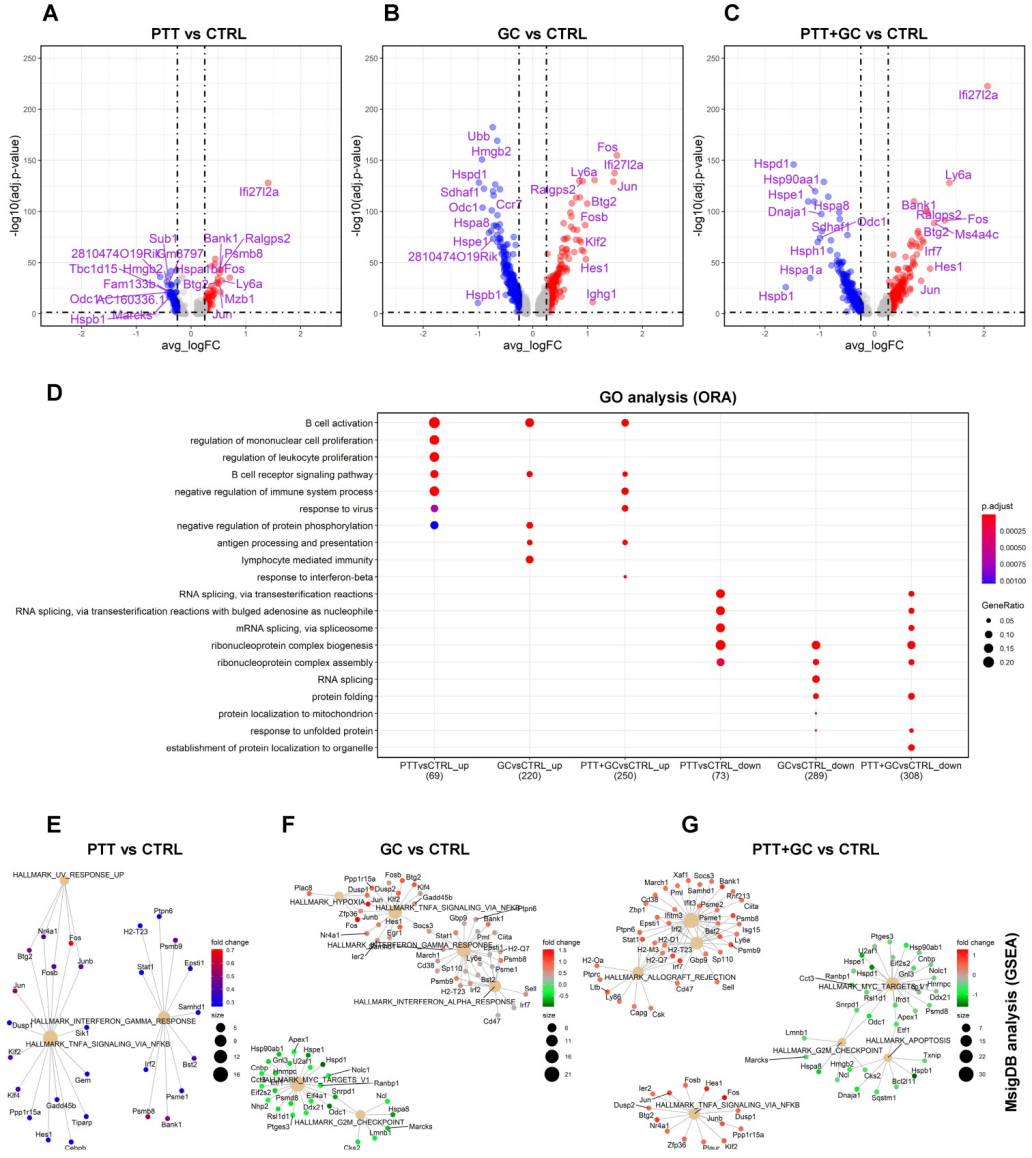
** Differential gene expression and pathway enrichment analysis for tumor-infiltrating B cells. (A)** Volcano plot showing differential gene expression comparing PTT with CTRL (PTT vs CTRL). Top 10 upregulated and downregulated genes are labeled. **(B)** Volcano plot showing differential gene expression comparing GC with CTRL. **(C)** Volcano plot showing differential gene expression comparing PTT+GC with CTRL. **(D)** Dot plot showing biological process (BP) of gene ontology (GO) analysis using over-representation analysis (ORA) method for both upregulated (up) and downregulated (down) genes for PTT vs CTRL, GC vs CTRL, and PTT+GC vs CTRL. **(E)** Network plot showing gene set enrichment analysis (GSEA) for DEGs from PTT vs CTRL using MsigDB hallmark gene sets. **(F)** Network plot showing the GSEA for DEGs from GC vs CTRL. **(G)** Network plot showing the GSEA for DEGs from PTT+GC vs CTRL.

ScRNA-seq technology not only dissects the cell heterogeneity but also captures the transcriptome of individual cells, expanding the broadness of researches in cell lineage, development, physiology, pathology and therapy 23-27. To explore the effect of each treatment on the functions of TIBs at the transcriptional level, we carried out differential gene expression and functional enrichment analysis. Three comparisons were made: PTT vs CTRL, GC vs CTRL, and PTT+GC vs CTRL. For each comparison, differentially expressed genes (DEGs) were generated by using *FindMarkers* function in *Seurat* R package 22. DEGs from comparing two treatment groups were defined as log Fold change > 0.25 or < -0.25 along with adjusted *p* value < 0.05, shown as red and blue dots in the volcano plots (Figure 2A-C). Functional enrichment analyses were performed using both over-representation analysis (ORA) 28 and gene set enrichment analysis (GSEA) 29. The *clusterProfiler* R package 30 was used to perform functional enrichment analysis for pseudotime-dependent genes by using ORA method and databases of Gene Ontology (GO) 31, Kyoto Encyclopedia of Genes and Genomes (KEGG) 32, Reactome Pathway Database (Reactome) 33 and Molecular Signatures Database (MsigDB) 34.

For PTT vs CTRL, 73 upregulated and 81 downregulated genes were shown in the volcano plot (Figure 2A). Biological process (BP) of gene ontology (GO) analysis using the ORA method demonstrated that PTT enriched B cell activation and B cell receptor (BCR) signaling pathways compared to CTRL (Figure 2D). This result was confirmed by KEGG analyses (Figure S2A) and Reactome analyses (Figure S2B). In contrast, PTT downregulated genes involved in RNA splicing and ribonucleoprotein complex biogenesis which likely affect protein translation (Figure 2D). KEGG and Reactome enrichment analyses gave similar results (Figure S2A-B).

GC stimulation upregulated 233 genes and downregulated 308 genes compared to CTRL (Figure 2B). GO analysis revealed that GC upregulated genes were involved in B cell activation, BCR signaling pathway, negative regulation of protein phosphorylation, antigen processing and presentation, and lymphocyte mediated immunity (Figure 2D, Figure S2A-B). Similar results were also obtained by using KEGG and Reactome analyses, including increases in expression of genes associated with the BCR signaling pathway and antigen processing and presentation (Figure S2A-B). Conversely, GC also downregulated genes involved in ribonucleoprotein complex biogenesis, RNA splicing, and protein folding (Figure 2D, Figure S2A-B).

When comparing PTT+GC vs CTRL, we observed 264 upregulated and 327 downregulated genes (Figure 2C). PTT+GC induced transcription of genes involved in B cell activation, BCR signaling, response to virus, antigen processing and presentation, and response to interferon beta (Figure 2D, Figure S2A-B). PTT+GC also downregulated genes involved in RNA splicing, ribonucleoprotein complex biogenesis, and protein folding (Figure 2D, Figure S2A-B).

The ORA analysis revealed that PTT, GC and PTT+GC initiated overlapping gene expression signatures (Figure 2D), although the numbers and fold changes of DEGs were not identical (Figure 2A-C). To further explore gene expression differences induced by these treatments, we used hallmark gene sets of MsigDB and conducted GSEA to visualize gene-pathway networks (Figure 2E-G). As shown in Figure 2E, PTT elevated IFNγ and TNFα signaling pathways. GC enriched IFNγ and TNFα signaling whilst downregulating Myc targets pathway (Figure 2F). PTT+GC upregulated IFNγ signaling, TNFα signaling, and uniquely enriched IFNα signaling. Like GC, PTT+GC also downregulated Myc targets and G2M checkpoint signaling (Figure 2G). These analyses showed that PTT, GC and PTT+GC had overlapping pathways including the IFNγ and TNFα responses. However, GC and PTT+GC enriched pathways had more associated genes than those enriched by PTT alone.

In addition to the MsigDb collection, the KEGG and Reactome pathway databases were used in GSEA. For PTT vs CTRL, no gene set enrichment was found, likely due to the small number of DEGs between the two groups. GC stimulation upregulated pathways involved BCR (Figure S2C) and IFNα signaling (Figure S2E) and downregulated spliceosome (Figure S2C) and mRNA splicing (Figure S2E) pathways. PTT+GC synergized to upregulate antigen processing and presentation pathways (Figure S2D) and IFNαβ signaling (Figure S2F), and downregulated spliceosome (Figure S2D) and heat shock protein composed pathways (Figure S2F). Taken together, both ORA and GSEA achieved consistent enrichment results (Figure 2D-G, S2A-F). It also demonstrated that treatments of PTT, GC, and PTT+GC had overlapping and nonoverlapping functions on B cells. This was further elucidated by the degrees of B cell activation from individual treatments to the combined PTT+GC treatment (Figure 2E-G, S2C-F). These results provide evidence that PTT+GC has a greater impact on activating B cell gene signatures and potential antitumor related functions than PTT or GC alone.

### Overlapping of treatment-upregulated genes in TIB cells

**Figure 3:**
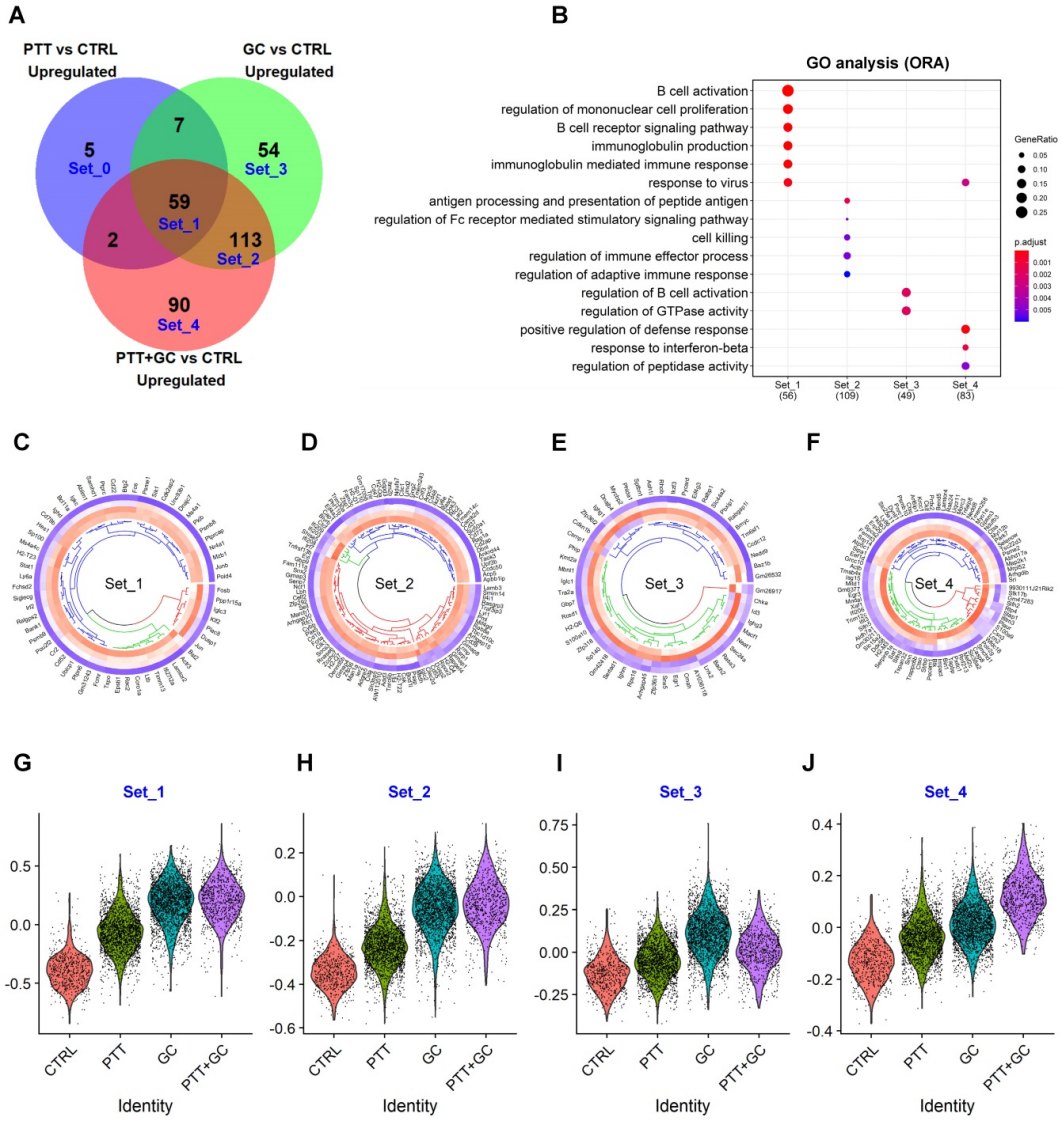
** Overlapping of treatment-upregulated genes. (A)** Venn diagram showing upregulated genes from comparisons of PTT vs CTRL, GC vs CTRL, and PTT+GC vs CTRL. Five gene sets, from Set_0 to Set_4, are labeled. **(B)** Dot plot for BP of GO analysis of genes in Set_1 to Set_4. **(C)** Circular heatmap showing the expression of genes from upregulated set 1 (Set_1) in each treatment group. Heatmap columns for groups of CTRL, PTT, GC and PTT+GC were arranged from outside to inside. Higher expression was colored in red while lower in blue. **(D)** Circular heatmap showing the expression of genes from upregulated set 2 (Set_2) in each treatment group. **(E)** Circular heatmap showing the expression of genes from upregulated set 3 (Set_3) in each treatment group. **(F)** Circular heatmap showing the expression of genes from upregulated set 4 (Set_4) in each treatment group. **(G)** Violin plot showing relative gene signature scores from Set_1 in each treatment group. **(H)** Violin plot showing relative gene signature scores from Set_2 in each treatment group. **(I)** Violin plot showing relative gene signature scores from Set_3 in each treatment group. **(J)** Violin plot showing relative gene signature scores from Set_4 in each treatment group.

We hypothesized that understanding the relationships among the treatment-regulated DEGs would shed light on the molecular mechanism stimulated by PTT+GC therapy. To determine B cell gene expression signatures associated with PTT+GC-enhanced survival of tumor-bearing mice, we investigated the relationship of treatment-regulated DEGs and focused on the PTT+GC-specific DEGs in TIBs. A Venn diagram was used to display the overlap of PTT, GC, PTT+GC regulated genes (Figure 3A). Genes uniquely regulated by PTT alone were confined in Set0. PTT-GC-(PTT+GC) intersecting genes were confined in Set1; GC-(PTT+GC) intersecting genes (excluding Set1) were confined in Set2. Genes uniquely regulated by GC alone were grouped in Set3. Similarly, Set0, Set3 and Set4 were defined as specific upregulation by PTT, GC and PTT+GC, respectively.

Collectively, 59 genes of Set1 were upregulated among PTT, GC, and PTT+GC (Figure 3A). Individually, PTT, GC and PTT+GC specifically upregulated 5 (Set0), 54 (Set3) and 90 (Set4) genes, respectively (Figure 3A). GO analysis for the individual gene sets were shown in Figure 3B. Biological processes (BP), such as B cell activation, BCR signaling, and response to virus were enriched in gene Set1 which were common to all 3 treatments (Figure 3B). Antigen processing and presentation of peptide antigens, cell killing, and regulation of immune effector processes were enriched in Set2, only shared by GC and PTT+GC (Figure 3B). Of note, Set2 contains more genes associated with class II antigen presentation machinery than Set1, suggesting that the difference between GC and PTT is that GC can activate more antigen-presenting processing genes than PTT. GC alone (Set3) specifically enriched pathways regulating B cell activation and GTPase activity (Figure 3B). PTT+GC-specific (Set4) pathways were enriched for positive regulation of the defense response and response to IFNβ (Figure 3B).

To confirm the above enrichment analysis for different gene sets, MsigDB, KEGG, and Reactome analyses were used. Similar enrichments were found (Figure S3A-C) compared with GO analysis (Figure 3B). For example, PTT+GC-specific genes (Set4) were involved with IFNγ and IFNα responsiveness (Figure S3A) and antigen processing and presentation (Figure S3B).

Although enrichments from genes in Set1 to Set4 share B cell activation related pathways, we hypothesized that their capacities for activating B cells were different for each treatment. To test this hypothesis, we compared the expression levels of each component of these confined gene sets in different treatment groups using circular heatmap. Set0 was not used for analysis likely due to the small number of genes. Even though Set1 genes were common to all treatment groups, genes with the highest level of expression were largely found in the PTT+GC group (Figure 3C). This was also observed in gene Set2 (shared by GC and PTT+GC) for selected genes (Figure 3D), again indicating that PTT+GC has a greater capacity for activating B cells than GC alone. Consistent with the pattern established in Set1 and Set2, Set4 genes showed the highest expression on B cells in the PTT+GC treated tumors (Figure 3F). In GC-specific upregulated gene Set3, GC treatment showed the highest promotion (Figure 3E).

Instead of showing the individual expression of genes from Set1 to Set4 in each treatment, we next used *AddModuleScore* function from *Seurat* R package 22 to calculate the average expression level of these upregulated gene sets. These expression scores for each treatment were displayed using violin plots with each dot representing a score of an individual cell (Figure 3G-J). The expression scores in gene Set1 (Figure 3G) and Set2 (Figure 3H) for GC and PTT+GC treatments were higher than that in CTRL and PTT, indicating that (1) Both PTT and GC upregulated B cell activation signatures compared with CTRL; (2) GC mainly promoted the degree of B cell activation signatures in Set1 when combining with PTT. GC has the highest B cell activation degree in Set3 (Figure 3I) because this gene set is GC-specifically upregulated. Consistently, PTT+GC has the highest B cell activation degree in Set4 because this gene set is PTT+GC-specifically upregulated (Figure 3J), also indicating that PTT and GC can synergize to promote the expression of gene signatures in Set4. This result is consistent with that of the gene expression dot plots (Figure 3C-F) and consistent with the hypothesis that although genes in Set1, 2, and 4 were all enriched in B cell activation related pathways, PTT+GC resulted in higher degrees of B cell activation than GC or PTT alone.

We further dissected the PTT+GC-specific upregulated genes by displaying several representative genes based on previous enrichment analysis and literature searches. We compared the gene expression among B cells after different treatments using dot plots (Figure S3D). It is worth noting that only PTT+GC vs CTRL, but not PTT vs CTRL or GC vs CTRL, resulted in significant expression changes in the indicated genes (adjusted *p* value < 0.05). We observed that PTT+GC-specific upregulated genes included Toll-like receptor cascades (Figure S3D). Among these genes, PTT+GC-specifically upregulated *Btk* and *Ctss*, which are not only involved in TLR signaling cascades, but also participate in antigen presentation. Additionally, PTT+GC-specific upregulated genes also included interferon signaling genes, such as *Ifit3*, *Ifitm3*, *Isg15*, *Xaf1*, *Ddx58*, *Nedd8*, *Rnf213*, *Tmsb4x*, *Trim12c* (Figure S3D). We also observed that PTT+GC upregulated cell survival genes, such as *Dynll1*, *Eef1d*, *Tnfaip8*, *Pecam1*, and favorable clinical prognosis genes, such as *Bin1*, *Erp29*, *Serpinb1a* (Figure S3D). The functions of these genes are listed in Table S1.

### Overlapping of treatment-downregulated genes in TIB cells

**Figure 4:**
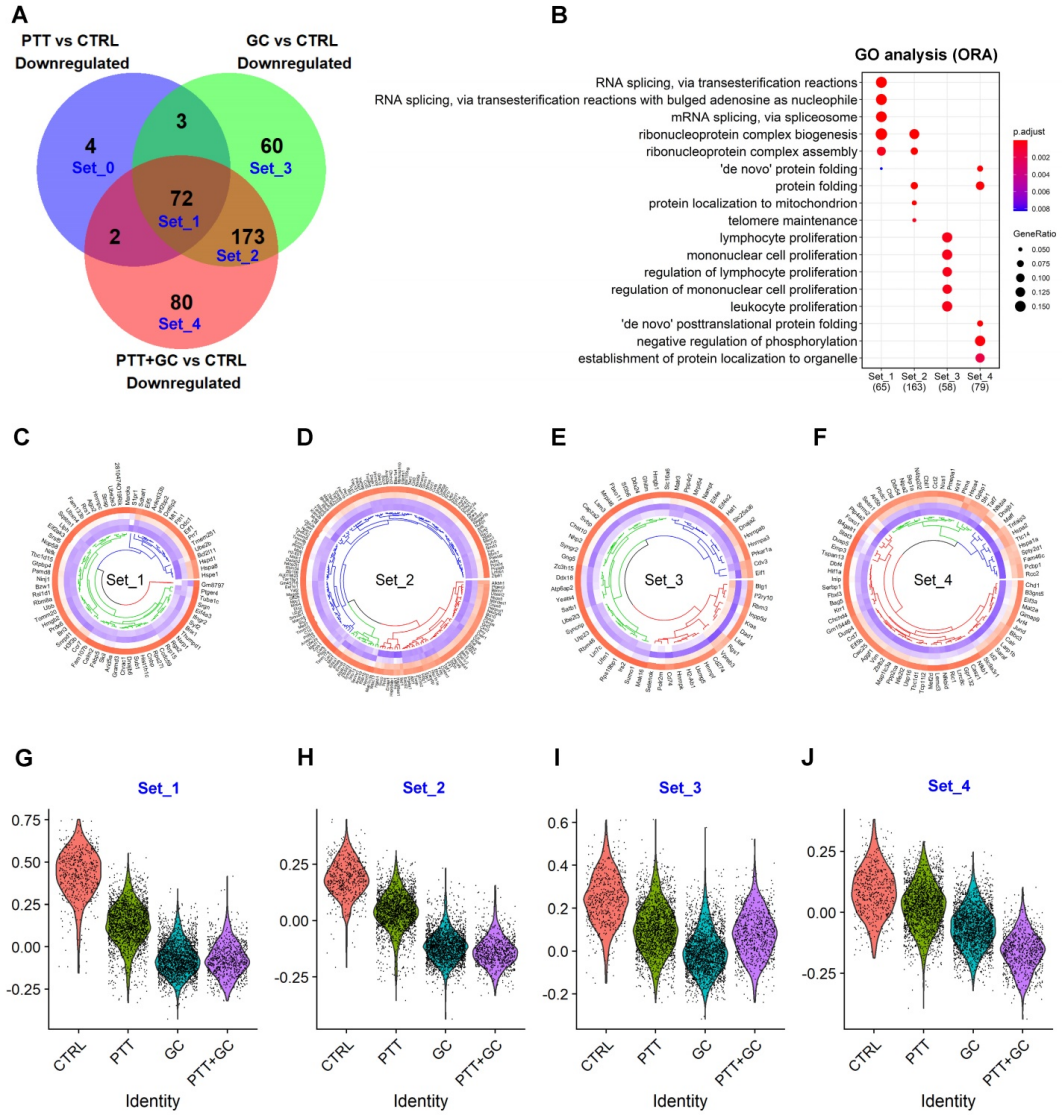
** Overlapping of treatment-downregulated genes. (A)** Venn diagram showing downregulated genes from comparisons of PTT vs CTRL, GC vs CTRL and PTT+GC vs CTRL. Five gene sets, from Set_0 to Set_4, were labeled. **(B)** Dot plot for BP of GO analysis of genes in Set_1 to Set_4. **(C)** Circular heatmap showing the expression of genes from downregulated set 1 (Set_1) in each treatment group. Heatmap columns for groups of CTRL, PTT, GC and PTT+GC were arranged from outside to inside. Higher expression was colored in red while lower in blue. **(D)** Circular heatmap showing the expression of genes from downregulated set 2 (Set_2) in each treatment group. **(E)** Circular heatmap showing the expression of genes from downregulated set 3 (Set_3) in each treatment group. **(F)** Circular heatmap showing the expression of genes from downregulated set 4 (Set_4) in each treatment group. **(G)** Violin plot showing relative gene signature scores from Set_1 in each treatment group. **(H)** Violin plot showing relative gene signature scores from Set_2 in each treatment group. **(I)** Violin plot showing relative gene signature scores from Set_3 in each treatment group. **(J)** Violin plot showing relative gene signature scores from Set_4 in each treatment group.

We next investigated the relationship of treatment-downregulated DEGs and focused on the PTT+GC-specific negative regulation on these genes in TIBs. A Venn diagram was used to illustrate the overlap of PTT, GC, and PTT+GC downregulated genes (Figure 4A). Genes intersected with different treatments were grouped similarly to Figure 3A: Set0 was for PTT-specific genes; Set1 for PTT-GC-(PTT+GC) intersecting genes; Set2 for GC-(PTT+GC) intersected genes (Set1 not included), Set3 for GC-specific genes, and Set4 for PTT+GC-specific genes (Figure 4A).

Collectively, for Set1, PTT, GC and PTT+GC commonly downregulated 72 genes (Figure 4A). GC and PTT+GC (Set2) co-downregulated 173 genes, while individually, PTT downregulated 4 genes (Set0), GC downregulated 60 genes (Set3), and PTT+GC downregulated 80 genes (Figure 4A). GO analysis of genes from the different sets was shown in Figure 4B. RNA splicing and ribonucleoprotein complex biogenesis and assembly pathways were enriched in Set1. Set2 was also enriched in genes involved in ribonucleoprotein complex biogenesis and assembly (Figure 4B) but with a greater number of genes involved in these pathways. GC specifically enriched for genes involved in the regulation of lymphocyte and mononuclear cell proliferation, while PTT+GC-specific genes were involved in protein folding, negative regulation of phosphorylation, and protein localization to organelles (Figure 4B). The results in Figure 4A-B demonstrate that PTT, GC and PTT+GC treatments shared some overlapping gene regulation, like what was observed for the upregulated genes in Figure 3. Using MsigDB (Figure S4A), KEGG (Figure S4B) and Reactome (Figure S4C) databases revealed similar findings.

Although RNA splicing and protein folding related pathways were shared among genes in Set1 and Set4, we assume their capacities for negatively regulating activated B cells were different in and unique to each treatment. To test this assumption, we compared the expression levels of these confined gene sets in different treatment groups using circular heatmap analysis. Set0 was not used for analysis likely due to the small number of genes. Gene expression pattern showed an increasing inhibition from CTRL to PTT, GC and PTT+GC in Set 1, 2 and 4, suggesting the unique and combinatory effect of each treatment in regulating these gene expression profiles (Figure 4C, 4D, 4F). In Set3, GC showed the highest inhibition due to these genes were GC-specific downregulated genes (Figure 4E).

Next, we calculated the average expression level of these downregulated genes from Set1 to Set4 in each treatment (Figure 4G-J). The expression scores for gene Set1 (Figure 4G) and Set2 (Figure 4H) were lower in GC and PTT+GC than that in CTRL and PTT, indicating that the degree of RNA splicing and potential negative regulation on B cell activations from Set1 and Set2 genes were predominantly inhibited by GC. GC showed the lowest downregulation on expression score in Set3 (Figure 4I) while PTT+GC exhibited the lowest score in Set4 (Figure 4J).

To further investigate the synergy between PTT and GC in terms of gene downregulation, we compared B cell gene expression among the four treatments using dot plots (Figure S4D). We found that selected PTT+GC-specific downregulated genes were involved in negative regulation of antigen-presenting, restriction of IFN production, cell death, and unfavorable clinical prognosis (Figure S4D). PTT+GC synergistically repressed genes, including *Aggf1*, *Calr*, *Cct2*, *Cct7*, *Chd1*, *Eif5b*, *Pmepa1*, *Rcc2*, *Serbp1*, *Skp1a*, *Slc9a3r1*, and *Vim* (Table S1), have been reported to be associated with unfavorable clinical prognosis, suggesting that PTT+GC may attenuate poor clinical prognosis and outcome.

### Single-cell trajectory analysis of TIBs reveals pseudotime progression driven by GC treatment

**Figure 5:**
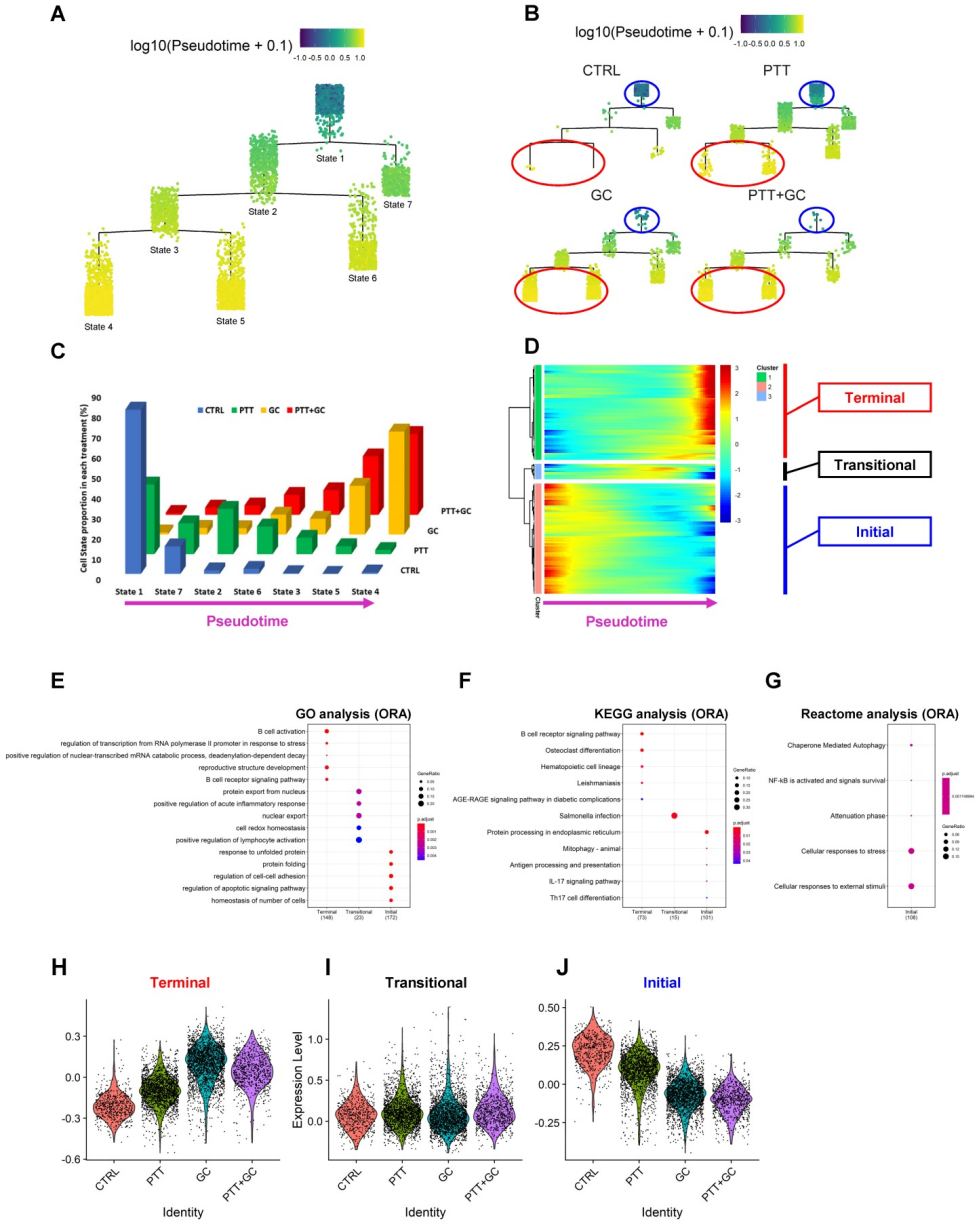
** Identification of GC/PTT+GC driven cell states, transcriptional signatures, and functional enrichment, by single-cell trajectory inference. (A)** Cell states following pseudotime in B cell trajectory. Pseudotime progresses from state 1 to states 2/7 to states 3/6 and finally to states 4/5. **(B)** Cell states in B cell trajectory for different treatment groups. Blue circles showed state 1 and red circles showed states 4 and 5. **(C)** Three-dimensional column plot showing distribution of cells in different states for each treatment group. Horizontal axis represents pseudotime progression. In CTRL and PTT groups, there is a decreasing pattern of cell state proportions, while in GC and PTT+GC groups there is an increasing pattern of cell state proportions. **(D)** Heatmap showing expression of genes along the pseudotime progression from left (state 1) to right (states 4/5). Genes were classified into 3 clusters according to their expression dynamics. Genes with expression patterns of “Terminal” (red), “Transitional” (black) and “Initial” (blue) are labeled. **(E)** Dot plot of the GO analysis using ORA method for genes with patterns of “Terminal”, “Transitional” and “Initial” along pseudotime progression. **(F)** Dot plot of the KEGG analysis using ORA method for genes with patterns of “Terminal”, “Transitional” and “Initial” along pseudotime progression. **(G)** Dot plot of Reactome analysis using ORA method for genes with patterns “Terminal”, “Transitional” and “Initial” along pseudotime progression. Only enrichment for “Initial” pattern was obtained. **(H)** Violin plot showing gene signature scores for “Terminal” genes in each treatment group. **(I)** Violin plot showing gene signature scores for “Transitional” genes in each treatment group. **(J)** Violin plot showing gene signature scores for “Initial” genes in each treatment group.

To further explore the treatment effects on TIB cell composition and function, we performed single-cell trajectory inference using *Monocle2* 35. The single-cell trajectory for TIBs was constructed with 7 states (branches) along with a progressing pseudotime (Figure 5A). CTRL and PTT-treated tumors contained more B cells than GC and PTT+GC in state 1 while GC- and PTT+GC-treated tumors contained more B cells in state 4 and state 5 than CTRL and PTT (Figure 5B). The pseudotime for the B cell trajectory progressed from state 1 (the root branch on the top) to state 4 and 5 (the terminal branches at the bottom), showing a clear transition of cell states from beginning state 1 to the ending states 4 and 5 induced by GC and PTT+GC (Figure 5B). Therefore, we established a connection between the pseudotime progression of B cells and the effect of GC treatment.

Next, we calculated B cell proportions for each state under different treatments. In CTRL group, 81% (459 out of 566) of B cells were assigned to state1 (Figure 5C). Along the pseudotime progression (state 1 to states 7/2 to states 6/3 and to states 5/4), the CTRL B cell proportion decreases significantly (Figure 5C). In the PTT group, most of the cells were with state 1, state 7 and state 2, corresponding with a cell proportion of 34%, 15% and 22%, respectively. The cell proportion in PTT showed a decreasing pattern along pseudotime progression, which was similar to that of CTRL but with a smoother degree of decline compared to CTRL. Interestingly, the opposite trend was observed for GC and PTT+GC treatments with the majority of TIBs residing in state 4 and 5 (Figure 5C). For example, the cell proportion with state 4 and state 5 in GC and PTT+GC groups reached 75% and 69%, respectively. These results clearly showed that GC and/or PTT+GC strongly switched the cell states when compared with CTRL and PTT (Figure 5A-C).

We hypothesized that gene dynamics along the trajectory would reflect GC and/or PTT+GC's regulatory mechanism on B cell properties. To test this hypothesis, we used *Monocle2* R package 35 to analyze the pseudotime-dependent genes in TIBs. Modules of genes that co-vary along pseudotime were visualized in the heatmap (Figure 5D). These genes, along the pseudotime, exhibited 3 expression patterns (Figure 5D): (1) the genes in red on the right side of the heatmap exhibited a progressively increased gene expression pattern (“Terminal”), ending in states 4 and 5; (2) the genes in red on the left side of the heatmap were enriched in state 1 and progressively-decreased (“Initial”); and (3) genes with yellow color in the middle of the heatmap showed a transitional expression pattern (“Transitional”).

To explore the function of these pseudotime-dependent genes, these 3 patterns of gene sets were extracted for functional enrichment analysis. GO and KEGG enrichment analyses using ORA method for the genes upregulated late in pseudotime were enriched in B cell activation and B cell receptor signaling pathways (Figure 5E-F). Conversely, the downregulated genes were enriched in protein folding, regulation of cell-cell adhesion, protein processing in ER, and mitophagy pathways (Figure 5E-G). For “Transitional” genes, enrichment results included protein export from nucleus, and positive regulation of acute inflammation by GO analysis (Figure 5E) and Salmonella infection by KEGG analysis (Figure 5F). It is worth noting that KEGG analysis (Figure 5F) for downregulated genes includes “antigen processing and presentation”, in which only HSP gene transcripts were found. These results demonstrated that B cell activation gene signature progresses along pseudotime and correlates with the transition of cell states caused by GC and/or PTT+GC. This analysis demonstrates the ability of GC and/or PTT+GC in switching TIBs from an inactivated state to an activated phenotype.

Furthermore, we calculated the expression scores for these “Terminal”, “Transitional”, “Initial” pseudotime-dependent gene sets. In “Terminal” gene set, expression scores in GC and PTT+GC were higher than that in CTRL and PTT (Figure 5H). In “Initial” module, an opposite trend was observed (Figure 5J). However, there was no apparent change of the expression scores among the four groups for the “Transitional” module (Figure 5I). This analysis (Figure 5H-J) confirmed the above enrichment analyses (Figure 5E-G).

To further validate the relationship of gene expression profiles with cell state changes, we assessed the DEGs generated by comparing states 4/5 versus state 1. We carried out the differential gene expression and functional enrichment analyses using the same parameters as previously described. DEGs from comparing states 4/5 vs state 1 were shown in the volcano plot (Figure S5A). GO, KEGG, and Reactome enrichment analyses using ORA method showed that in states 4/5, TIBs were enriched in genes involved in B cell activation, response to type I IFN (Figure S5B), BCR signaling (Figure S5B-C), antigen processing and presentation (Figure S5C), and antigen processing-cross presentation (Figure S5D). This indicated that B cells in states 4/5 were more activated than those in state 1, correlating with the upregulated genes from the trajectory analysis (Figure 5E-G). Alternatively, RNA splicing (Figure S5B-D), protein folding/processing (Figure S5B-C), and heat shock protein (HSP) 90 related pathways (Figure S5D) were downregulated. In addition to ORA, we used GSEA to further analyze the gene enrichment conditions for these DEGs between state 4/5 and state 1. GSEA MsigDB analysis revealed that TIBs in states 4/5 expressed upregulated type I and type II IFN and TNFα pathways, with downregulated genes involved in Myc target pathways (Figure S5E). GSEA KEGG analysis revealed that BCR signaling pathway was enriched while spliceosome pathways were diminished (Figure S5F). Consistently, the Reactome database revealed the TIBs in states 4/5 expressed genes involved in IFNαβ signaling, cytokine signaling, with downregulated genes involved in autophagy and cellular stress (Figure S5G).

Taken together, the comprehensive gene functional enrichment analyses concluded that the GC and PTT+GC treatment induced a cell state change that correlated with B cell activation. This strongly suggests a molecular mechanism by which B cells play an important role in executing an anticancer effect directly or indirectly (by collaborating with other cell types) in the TME.

### Pro-inflammatory effect by PTT+GC is stronger than GC alone in subsets of differentiated B cell states

**Figure 6:**
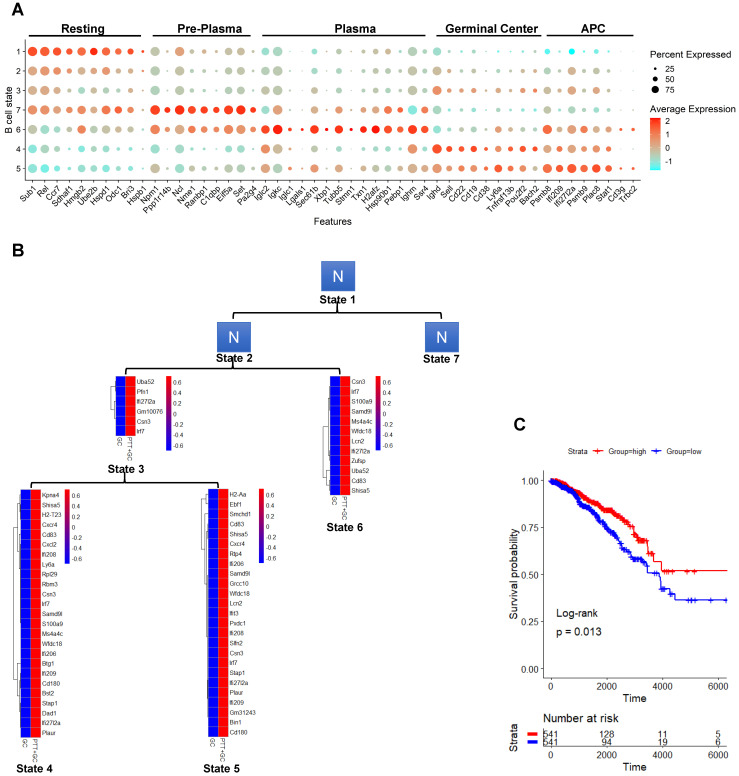
** Identification of B cell states, higher pro-inflammatory effect by PTT+GC versus GC, and association of PTT+GC specifically upregulated genes with breast cancer patient survival. (A)** Dot plot showing expression of B cell state marker genes. States were arranged along the pseudotime. B cells were identified as “Resting”, “Pre-Plasma”, “Plasma”, “Germinal Center” and “APC”. **(B)** Heatmaps showing the upregulated genes from comparison of PTT+GC vs GC in B cell states 3, 6, 4 and 5. These upregulated genes were not found in states 1, 2, and 7 (shown as N). **(C)** Kaplan-Meier plots showing the significant difference in survival time (days) between breast cancer patients in groups with “high” and “low” expressions of PTT+GC vs GC-derived upregulated genes from (B). Patient groups were stratified by the median of enrichment score calculated by gene set variation analysis (GSVA). Log-rank method was used for statistical analysis.

As GC drove the progression along pseudotime with shifting the cells from state 1 into states 4 and 5, we sought to define what type of B cell resides in each state. By finding enriched genes specific for each state, TIBs in state 1 were determined to be stimulated, yet undifferentiated B cells, compared to that in states 4 and 5. For example, B cells in state 1 expressed with genes involved in antigen presentation and processing, germinal center formation and homeostasis, and T cell activation and interaction (Figure 6A). These data suggest that the B cells were under the process of encountering antigen, but the TME confined B cell differentiation. Treatment with GC and PTT+GC released the restriction on B cells allowing them to differentiate toward effector subsets.

The B cells residing in states 2 and state 3 were characterized by few genes and unable to be defined. Likely these B cells were in a transitional state, on their way to becoming more differentiated effector B cells. State 7 was characterized as a plasma cell precursor (pre-plasma) (Figure 6A). Many of the genes characterizing this state were involved in cellular metabolism, transcription, translation, and protein folding, but no Ig genes were observed. However, further along the pseudotime progression, many Ig genes were observed in state 6, suggesting that these cells may be plasma cell-like. For example, B cells in state 6 expressed *Iglc2*, *Igkc*, *Iglc1*, and *Ighm*, genes encoding the constant region of immunoglobin light chains (Figure 6A). Further supporting this conclusion, B cells in state 6 express *Lgals1*, *Sec61b*, and *Xbp1*, all genes induced during plasma cell differentiation. Likely the cells in state 7 were plasma cell precursors and cells in state 6 are differentiating plasma cells.

Next, we characterized state 4 as germinal center-like B cells since they expressed *Ighd*/IgD^+^, *Sell*/CD62L^+^, CD22^+^, CD19^+^, CD38^+^, *Ly6a*/Sca-1^+^, and *Tnfrsf13b*/TACI^+^ (Figure 6A). Furthermore, state 4 B cells expressed the germinal center transcription factors *Pou2f2* (OCT2) and *Bach2*, and Ig genes required for antibody production. While state 4 had >100 genes characterizing this state, state 5 only had 24 transcripts. Cells in state 5 expressed genes involved in antigen processing, base excision and repair for class switch recombination, and express genes involved in T cell interaction such as *Plac8* and *Zpb1*. From this we concluded that state 5 B cells were memory-like B cells (Figure 6A).

As tumor growth was suppressed by PTT+GC, but not by GC alone (Figure 1A-B), we assessed the difference between GC and PTT+GC treatments by comparing the gene expression signatures of TIBs residing in states 3, 4, 5 and 6 (Figure 6B). A common theme among the PTT+GC treated cells residing in these states is a type I IFN signature, including *Bst2*, *Ifi206*, *Ifi208*, *Ifi209*, and *Ifit3*. The data suggest that these B cells have received type I IFN signals during their activation and differentiation (Figure 6B). Furthermore, upregulation of *Cxcr4* and *Cxcl2*, and *H2-Aa* by PTT+GC treatment indicates an increase in antigen presentation and pro-inflammatory functions of the tumor-infiltrating B cells. These upregulated genes prompted us to hypothesize that PTT+GC may stimulate a more robust pro-inflammatory response than GC alone (Figure 6B) and in turn lead to prolonged survival of tumor-bearing animals (Figure 1A-B).

We next determined if the efficacy of PTT+GC treatment, in mouse breast tumors, translates to human disease by determining the relationship between the expression level of the PTT+GC vs GC upregulated genes and clinical outcomes in breast cancer patients. We utilized these upregulated and downregulated genes to obtain the enrichment score (ES) based on gene set variation analysis (GSVA) 36 (Figure S6A). Breast cancer patients with a higher expression of the genes found in the PTT+GC samples exhibited prolonged survival compared to the patients with lower gene expression (Figure 6C). This demonstrated that the genes specifically induced by PTT+GC, but not GC alone (Figure 6B), correlated with greater overall survival in breast cancer patients. On the other hand, the PTT+GC vs GC derived downregulated genes did not affect patient survival (Figure S6B). Taken together, we provided predictive evidence that PTT+GC treatment modalities have a greater potential in prolonging patient survival by driving B cell activation.

## Discussion

Although it has been shown that tumor-infiltrating T cells participate in anti-tumor immune responses 37, there are increasing reports showing that TIBs have antitumor functions and indicate a favorable prognosis in many cancer types, including breast cancer 38. This beneficial effect of the TIBs was attributed to the following processes: (1) antibody production; (2) antigen presentation; (3) tumor-killing cytokine secretion 39, 40. Previously, we reported that a combination of locally administered PTT with GC induced systemic immunity against established tumors and metastases and prolonged animal survival in several aggressive tumor models 41-43. To further understand the mechanism of localized ablative immunotherapy (LAIT), we applied scRNA-seq analysis to investigate the functional changes in the tumor-infiltrating leukocytes, particularly B cells, in the TME after PTT+GC treatment.

Specifically, we adopted LAIT (Figure S1) and successfully extended the survival of mice bearing MMTV-PyMT mammary tumors (Figure 1A-B). LAIT increased the TIB proportions (Figure 1G-H), and induced expression of genes enriched in B cell activation, including antigen-presentation (Figure 2D, S2A-B) and type I interferon signaling (Figure 2D, 2G, S2F, 3B, S3A). Single-cell trajectory analysis, which represents pseudotime progression, showed that both PTT+GC and GC, but not PTT, induced activation and differentiation of resting B cells toward an effector phenotype (Figure 5A-F, 6A). It also revealed that the B cells in the activated states were enriched with antitumor related gene expression (Figure 5E-J), specifically by PTT+GC treatment. We also showed that the combination of PTT and GC, not GC alone, induced higher B cell activation (Figure 6B) and upregulated genes associated with greater overall survival in breast cancer patients (Figure 6C).

We previously reported that, based on imaging observation and analysis, neutrophil infiltration was induced by the combination therapy of locally administered PTT with GC against B16 melanoma 41. However, whether B cells were affected by LAIT treatment was not known. In this work, using scRNA-seq analysis, which can dissect both the cell heterogeneity and transcriptional changes, we found B cells constituted 12% of all CD45^+^ immune cells, much more than that of neutrophils/granulocytes (3%). That prompted us to investigate the potential regulations of LAIT on this presumed humoral immunity and antibody-producing related cells. In this experiments, LAIT-treated MMTV-PyMT tumors exhibited increased proportions of TIBs, enriched for the expression of genes involved in B cell activation, antigen-presenting, and IFNαβ signaling responses. Although PTT+GC did not result in quantitatively or proportionally more tumor-infiltrating B cells when compared to PTT and GC alone, the protective anti-tumor pathways, including “positive regulation of defense response” and “response to interferon-beta”, were unique to PTT+GC (Figure 3B, F, J). Additionally, the expression level of these protective anti-tumor genes also showed the pattern of being the highest in PTT+GC, when compared with other groups (Figure S3D). Therefore, although “quantity” (number, proportion) and “quality” (gene expression) of immune cells are important factors in the tumor microenvironment, B cell “quality” is more important than “quantity” in inducing anti-tumor responses. Therefore, it is the functionality, not the number of B cells, after the LAIT treatment, that made the difference in efficacy. On the other hand, we did not find clearly defined plasma cells in the tumors 9 days after local treatments, single-cell trajectory analysis revealed that B cells residing in state 7 and state 6 (Figure 6A) were potential plasma-like precursor cells and plasma-like cells. Analysis of TIBs at a later time point may reveal the development of plasma cells within the TME following LAIT.

LAIT-activated TIBs amplified their APC function, as these cells were enriched in genes associated with antigen-presentation. Furthermore, both GC and PTT+GC upregulated the expression of *Cd40* and downregulated suppressing molecule *Cd274* (PD-L1) 44 in TIBs, but PTT+GC selectively induced CD40 ligand (*Cd40lg*) expression in conventional CD4T cells (Figure S1C), suggesting that LAIT treated TIBs could interact with tumor-infiltrating T helper cells and inhibit the suppressing molecule PD-L1 expression in B cells to further augment a potent antitumor immune response. Further supporting T-B cell interactions is the activation of type I IFN signature in B cells, which in turn drives T cell responses to eliminate tumors. Strikingly, this immunological cascade was reported to drive inflammation in certain autoimmune diseases that have increased expression of type I IFN inducible genes 45-47. This was best characterized in neuromyelitis optica, where type I IFNs directly acted on B cells to promote autoreactive T cell responses, which exacerbates inflammation of the central nervous system 47. While detrimental in autoimmunity, this cascade is beneficial for immune mediated cancer elimination.

Although both GC and PTT+GC induce the differentiation of TIBs, further comparison using single-cell trajectory analysis between these two groups revealed that PTT+GC induced a greater pro-inflammatory response in TIBs with more activated attributes (Figure 6B). Furthermore, PTT+GC specifically upregulated a set of genes that correlated with greater overall survival in breast cancer patients (Figure 6A). Genes specifically upregulated by GC alone (downregulated by PTT+GC) did not have this effect (Figure S6B). We hypothesize that PTT disrupted tumor homeostasis and released tumor-specific antigens. GC drove a type I IFN response, which in turn enhanced the antigen-presenting functions of TIBs. Synergizing the functions of PTT and GC led to a strong antitumor immunity, because PTT allows the immune stimulating function of GC to overcome the anti-inflammatory TME.

In our results, we found PTT+GC increased the cell proportion of TIBs and upregulated genes that have a positive association with the overall survival of breast cancer patients (Figure 6C). In support of this observation, a study on hepatocellular carcinoma (HCC) indicated that CD20^+^ TIBs were positively correlated with small tumor size and the increase of CD20^+^ TIBs was significantly correlated with the overall and recurrence-free survival rate of HCC patients 48. It was also reported that, in colorectal cancer, the high density of CD20^+^ TILs were significantly correlated with the improvement of overall survival of patients 49. Additionally, in muscle-invasive bladder cancer (MIBC), the overall survival of the patients with high CD19^+^ TIB MIBC was significantly longer and CD19**^+^** TIBs were identified as an independent prognostic factor and served as antigen-presenting cells (APCs) to activate CD4^+^ T cell in the TME 50. These data support our conclusion on the importance of B cells in cancer control. Furthermore, we speculate that patients who do not have the favorable B cell signatures could benefit from LAIT-induced B cell immune responses.

Altogether, this study demonstrates the effectiveness of LAIT in a breast cancer model. Our data provided novel insights into how LAIT-induced B cell activation contributes to the upregulation of pro-inflammatory genes that show positive correlation with prolonged patient survival. Overall, these findings broadened our understanding of LAIT's regulatory roles in remodeling TME and shed light on the potential of B cell activation in clinical applications.

## Methods

### Study approval

Experimental procedures and handling of mice were performed in accordance with the University of Oklahoma Health Sciences Center (OUHSC) and Oklahoma Medical Research Foundation (OMRF) Institutional Animal Care and Use Committee (IACUC) regulations and approved protocols.

### Mice

Female FVB/NJ wild-type (stock #001800) and FVB/N transgenic MMTV-PyMT (stock #002374) mice were purchased from Jackson Laboratories and housed according to institutional guidelines at the University of Oklahoma Health Sciences Center. All studies were approved and adhered to OUHSC Institutional Animal Care and Use Committee protocols (#17-032-HCL).

### Syngeneic tumor cell transplantation

MMTV-PyMT murine breast tumor organoids were isolated from FVB/N-Tg (MMTV-PyVT) 634M ul/J mice as previously described 21. Briefly, cells were incubated overnight in mammary epithelial cell media (DMEM/F12 supplemented with 10% fetal bovine serum (Sigma Aldrich, F2442-500ML) 100 U/mL penicillin-streptomycin (Sigma Aldrich, P0781-20×100 ml), 5ug/mL insulin-transferrin-selenium (ThermoFisher Scientific, 51500056), 1ug/mL hydrocortisone (Sigma Aldrich, H0888-10G), 10 ng/mL mouse epidermal growth factor (EGF) (ThermoFisher Scientific, 53003018), and 50 ug/mL gentamicin (Genesee Scientific, 25-533). Cells were washed twice with Hepes Buffered Saline (HBSS) (Gibco, 14175-095) trypsinized, and resuspended to a concentration of 1x10^5^ cell per 20 uL. Cells were injected into the mammary fat pad of wild type FVB mice without clearing. The incision was closed with Vetbond tissue adhesive (3M).

### Treatment of mouse tumors

When tumors reached 0.5 cm^3^, the tumor site was shaved and mice received one of four treatments: CTRL (without treatment), GC alone, PTT alone, and LAIT (PTT+GC). The mice in GC group received an intratumoral injection of 1% GC in 0.1 ml solution (Immunophotonics, Inc.). For mice in PTT group, the laser parameters were selected based on our previous studies 42, 51-54. The tumor was treated by an 805-nm laser (AngioDynamics, Latham, NY) with a power density of 1 W/cm^2^ for 10 minutes, using an optical fiber with a diffusion lens (Pioneer Optics, Bloomfield, CT) to delivery uniform light distribution on the treatment surface. For the mice in LAIT group, the tumor was treated by the laser (1 W/cm^2^ for 10 minutes), followed by an intratumoral injection of GC (0.1 ml at 1%). Ten days after treatment, tumors were collected from selected mice in each group. The remaining mice were observed for up to 60 days or when the tumors reached a size of 2.5 cm^3^ or reached ethical endpoints.

### Sample preparation and single-cell RNA sequencing library generation

In each of the four treatment groups, immune cells from four mice were used for single-cell RNA sequencing. Tumor tissues were isolated, minced with scalpels, and digested with Collagenase IV and Dnase I at 37°C for 20-30 minutes. After enzymatic digestion, immune cells were enriched using lymphocyte separation medium. The enriched cells were then subjected to magnetic bead separation (EasySep Mouse Streptavidin Rapidspheres Isolation Kit, Stem Cell, 19860) to remove the EpCAM^+^ cells (CD326 1:200, ThermoFisher Scientific, 13-5791-82). The EpCAM-depleted cells were stained with antibodies against CD45 and a viability dye. Live CD45^+^ cells (CD45 1:100, Biolegend, 103112), were sorted using MoFlo and then processed for droplet-based 3' end scRNA-seq by encapsulating sorted live CD45^+^ tumor-infiltrating immune cells into droplets via a 10× Genomics platform according to the manufacturer's instructions (10× Genomics). Paired-end RNA-seq was performed via an Illumina NovaSeq 6000 sequencing system.

### Data processing

The *Cell Ranger* pipeline was used to perform sample sequencing reads. The Linux command *cellranger count* was used to process sample-specific fastq files.* Seurat*-guided analyses were used to process and integrate datasets from different treatment groups 22, 55. Genes that were expressed in less than 5 cells or cells that expressed less than 800 and more than 6000 genes were excluded. Also, cells with a mitochondria percentage over 10% were excluded. Most variable genes were identified using the *FindVariableFeatures* function by setting feature numbers as 2000. Principal component analysis (PCA) was performed using the first 30 principal components (PCs). Clustering was performed using *FindClusters* that implements a shared nearest neighbor (SNN) modularity optimization-based clustering algorithm with resolution 0.5 for default analysis. B cells were subset and re-clustered to generate its t-SNE plot.

### Differential gene expression (DGE) analysis

Comparisons were conducted by comparing treatments of PTT, GC, and PTT+GC with CTRL, respectively. Differential gene expression was analyzed by using *FindMarkers* function in *Seurat* R package 22 for each comparison and visualized by volcano plot. Threshold of Log fold change (logFC) was set at 0.25 and -0.25 as default. Top 10 of both upregulated and downregulated differentially expressed genes (DEGs) were labelled on volcano plots.

### Functional enrichment analysis

*clusterProfiler* R package 30 was used for gene functional enrichment analysis. Both methods of over-representation analysis (ORA) and gene set enrichment analysis (GSEA) were used. Corresponding pathway databases include GO, KEGG, Reactome and MsigDB etc. Dot plot was mainly used for ORA result visualization and network plot for GSEA result.

### Analysis of overlapping of treatment-derived DEGs

Overlapping of treatment-derived DEGs were analyzed and visualized by using Venn Diagram. Subset of indicated gene sets were used for functional enrichment analysis. Expression of these subset genes were obtained using *AverageExpression* function in *Seurat* R package 22 and visualized using circular heatmap 56, 57.

### Analysis of correlation between clinical survival and derived gene sets

To assess the clinical significance of expression of a given gene set, we utilized gene expression RNA-seq and survival data available from GDC TCGA Breast Cancer (BRCA) (20 datasets) 58. To evaluate whether there was a relationship between differentially expressed gene sets and clinical survival in breast cancer patients, we utilized PTT+GC vs GC uniquely expressed gene sets as the input and get the enrichment score (ES) using gene set variation analysis (GSVA) R package 36. Cancer patients were considered “high” if ES was above the median and “low” if ES is below median. Analyses were carried out with Kaplan-Meier estimates and log-rank tests. Numbers below plots represent numbers of breast cancer patients. Survival times are presented as days.

### Single-cell trajectory analysis

*Monocle2* R package was used for single-cell trajectory inference based on the *DDRTree* algorithm 35. The construction of tumor-infiltrating B cell *Seurat* object was imported into *Monocle2* and highly variable genes were used for gene ordering and trajectory and pseudotime construction. Heatmap for pseudotime associated genes were generated and clusters of genes were selected for downstream functional enrichment analysis.

### Statistical analyses

Statistical analysis was conducted with GraphPad Prism and R. Unless indicated otherwise, data were expressed as means ± SD or SEM. One-way analysis of variance (ANOVA) was used for multiple group comparisons. Proportion test function (“prop.test”) in R was used for cell proportion test. Differences in survival were determined on the basis of Kaplan-Meier survival analysis. Adjusted *P* values less than or equal to 0.05 were considered statistically significant (*, *p* ≤ 0.05; **, *p* ≤ 0.01).

### Data and code availability

The accession number for the scRNA-seq data reported in this paper is GEO: GSE150675. Analysis for such data can be available upon request.

## Supplementary Material

Supplementary figures and table.

## Abbreviations

CTRL: control
PTT: photothermal therapy
GC: N-dihydrogalactochitosan
LAIT: localized ablative immunotherapy
MMTV-PyMT: mouse mammary tumor virus-polyoma middle tumor-antigen
scRNA-seq: single-cell RNA sequencing
TILs: tumor-infiltrating lymphocytes
TIBs: tumor-infiltrating B cells
TME: tumor microenvironment
IFN-γ: interferon-γ
TNFα: tumor necrosis factor-α
t-SNE: t-distributed stochastic neighbor embedding
DEGs: differentially expressed genes
ES: enrichment score
GO: gene ontology
ORA: over-representation analysis
GSEA: gene set enrichment analysis
GSVA: gene set variation analysis
KEGG: kyoto encyclopedia of genes and genomes
MsigDB: Molecular Signatures Database
