# Supplementary material for: Antigen presentation and interferon signatures in B cells driven by localized ablative cancer immunotherapy correlate with extended survival
Source: Theranostics. 2022 Jan 1;12(2):639–56. doi: 10.7150/thno.65773 (PMC8692917; doi:10.7150/thno.65773)
Supplement: Supplementary file 1 — Supplementary figures and table. [file thnov12p0639s1.pdf]

Supplemental Figure Legends

Figure S1. Schematic of treatment and analysis pipeline for tumor-infiltrating B cells (related to Figure 1).

(A) Schematic of MMTV-PyMT tumor implantation, treatment, and data analysis in mice.

(B) Schematic of analysis flowchart for tumor-infiltrating B cells. DGE: differential gene expression

(C) Dot plot showing the expression of *Cd40* in B cells, *Cd40lg* in conventional CD4T (CD4Tconv) cells, and *Cd274*(PD-L1) in B cells.

Figure S2. Differential gene expression and pathway enrichment analysis for tumor-infiltrating B cells (related to Figure 2).

(A) Dot plot showing KEGG enrichment analysis using ORA method for both upregulated and downregulated genes from comparisons of PTT vs CTRL, GC vs CTRL and PTT+GC vs CTRL.

(B) Dot plot showing Reactome enrichment analysis using ORA method for both upregulated and downregulated genes from comparisons of PTT vs CTRL, GC vs CTRL and PTT+GC vs CTRL.

(C) Network plot of the KEGG enrichment analysis using GSEA method for DEGs from GC vs CTRL.

(D) Network plot of the KEGG enrichment analysis using GSEA method for DEGs from PTT+GC vs CTRL.

(E) Network plot of the Reactome enrichment analysis using GSEA method for DEGs from GC vs CTRL.

(F) Network plot of the Reactome enrichment analysis using GSEA method for DEGs from PTT+GC vs CTRL.

Figure S3. Overlapping of treatment-upregulated genes (related to Figure 3).

(A) Dot plot for MsigDB enrichment analysis of genes in Set_1 to Set_4 (enrichment of Set_3 not found).

(B) Dot plot for KEGG enrichment analysis of genes in Set_1 to Set_4 (enrichment of Set_3 not found).

(C) Dot plot for Reactome enrichment analysis of genes in Set_1 to Set_4 (enrichment of Set_3 and Set_4 not found).

(D) Dot plot of the expression of PTT+GC-specific upregulated genes in Set_4. These genes are involved in pathways of TLR, APC, IFN, cell survival, favorable prognosis, and metabolism (Table S1).

Figure S4. Overlapping of treatment-downregulated genes (related to Figure 4).

(A) Dot plot for MsigDB enrichment analysis of genes in Set_1 to Set_4.

(B) Dot plot for KEGG enrichment analysis of genes in Set_1 to Set_4.

(C) Dot plot for Reactome enrichment analysis of genes in Set_1 to Set_4 (enrichment of Set_3 not found).

(D) Dot plot of the expression of PTT+GC-specific downregulated genes (Set_4). These genes are involved in pathways of negative regulation of APC, IFN, cell death and prognosis (Table S1).

Figure S5. Identification and enrichment analysis of differentially expressed genes in B cells from comparing activated cell states (states 4 and 5) with inactivated (state 1) (related to Figure 5).

(A) Volcano plot for both upregulated and downregulated differentially expressed genes (DEGs) from comparison of activated cell states (states 4 and 5) with inactivated state (state 1). Top 10 upregulated and downregulated genes are labeled.

(B) Dot plot of the GO enrichment analysis for DEGs using ORA method.

(C) Dot plot of the KEGG enrichment analysis for DEGs using ORA method.

(D) Dot plot of the Reactome enrichment analysis for DEGs using ORA method.

(E) Network plot of MsigDB hallmark gene sets enrichment analysis for DEGs using GSEA method.

(F) Network plot of KEGG enrichment analysis for DEGs using GSEA method.

(G) Network plot of Reactome enrichment analysis for DEGs using GSEA method.

Figure S6. Association of PTT+GC vs GC-derived downregulated genes with breast cancer patient survival (related to Figure 6).

(A) Flowchart for analyzing overall survival of breast cancer patient using GSVA method.

(B) Kaplan–Meier plots showing the insignificant difference in survival time (days) between breast cancer patients in groups with "high" and "low" expression of PTT+GC vs GC-derived downregulated genes. Patient groups were stratified by the median of enrichment score calculated by GSVA. Log-rank method was used for statistical analysis.

Table S1. Collection of reported functions for PTT+GC specifically upregulated and downregulated genes.

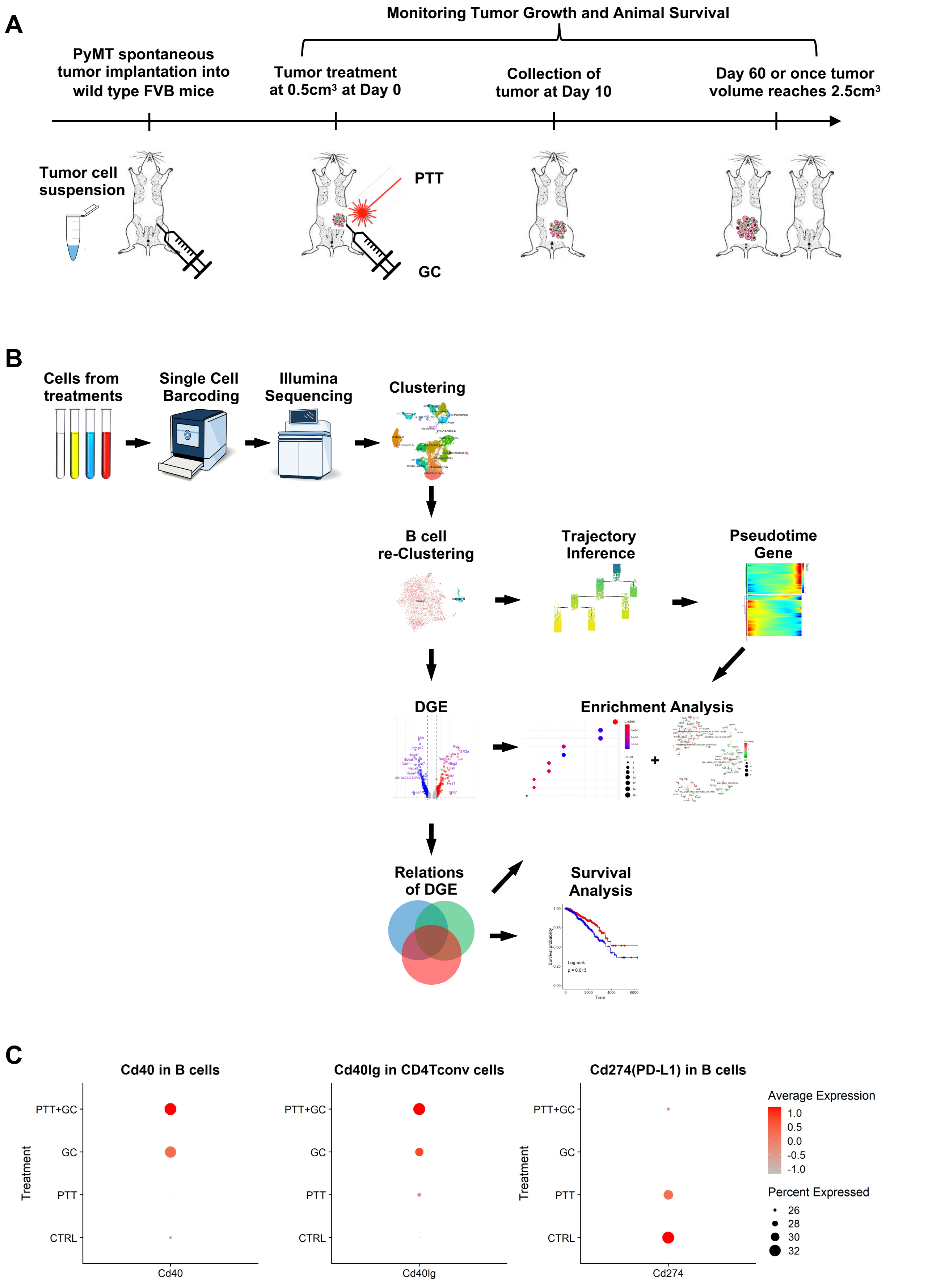
A
Monitoring Tumor Growth and Animal Survival
PyMT spontaneous tumor implantation into wild type FVB mice
Tumor treatment at 0.5cm³ at Day 0
Collection of tumor at Day 10
Day 60 or once tumor volume reaches 2.5cm³
Tumor cell suspension
PTT
GC
B
Cells from treatments
Single Cell Barcoding
Illumina Sequencing
Clustering
B cell re-Clustering
Trajectory Inference
Pseudotime Gene
DGE
Enrichment Analysis
Relations of DGE
Survival Analysis
C
Cd40 in B cells
Cd40lg in CD4Tconv cells
Cd274(PD-L1) in B cells
Treatment
PTT+GC
GC
PTT
CTRL
Cd40
Cd40lg
Cd274
Average Expression
1.0
0.5
0.0
-0.5
-1.0
Percent Expressed
26
28
30
32

Figure S1.

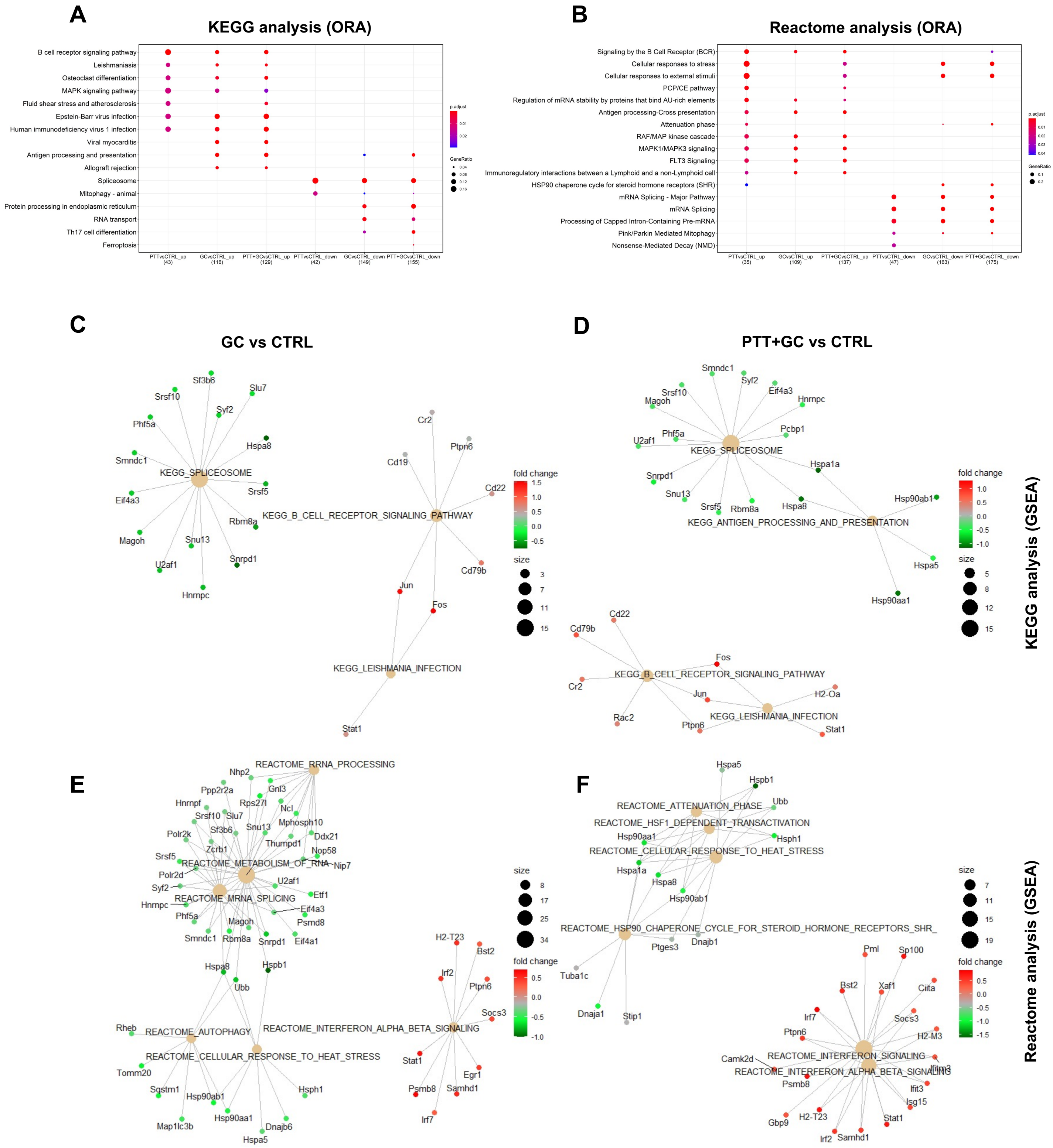

A
KEGG analysis (ORA)
B cell receptor signaling pathway
Leishmaniasis
Osteoclast differentiation
MAPK signaling pathway
Fluid shear stress and atherosclerosis
Epstein-Barr virus infection
Human immunodeficiency virus 1 infection
Viral myocarditis
Antigen processing and presentation
Allograft rejection
Spliceosome
Mitophagy - animal
Protein processing in endoplasmic reticulum
RNA transport
Th17 cell differentiation
Ferroptosis
PTTvsCTRL_up (43)
GCvsCTRL_up (116)
PTT+GCvsCTRL_up (129)
PTTvsCTRL_down (42)
GCvsCTRL_down (149)
PTT+GCvsCTRL_down (155)
p.adjust
GeneRatio
B
Reactome analysis (ORA)
Signaling by the B Cell Receptor (BCR)
Cellular responses to stress
Cellular responses to external stimuli
PCP/CE pathway
Regulation of mRNA stability by proteins that bind AU-rich elements
Antigen processing-Cross presentation
Attenuation phase
RAF/MAP kinase cascade
MAPK1/MAPK3 signaling
FLT3 Signaling
Immunoregulatory interactions between a Lymphoid and a non-Lymphoid cell
HSP90 chaperone cycle for steroid hormone receptors (SHR)
mRNA Splicing - Major Pathway
mRNA Splicing
Processing of Capped Intron-Containing Pre-mRNA
Pink/Parkin Mediated Mitophagy
Nonsense-Mediated Decay (NMD)
PTTvsCTRL_up (35)
GCvsCTRL_up (109)
PTT+GCvsCTRL_up (137)
PTTvsCTRL_down (47)
GCvsCTRL_down (163)
PTT+GCvsCTRL_down (175)
p.adjust
GeneRatio
C
GC vs CTRL
KEGG_SPLICEOSOME
KEGG_B_CELL_RECEPTOR_SIGNALING_PATHWAY
KEGG_LEISHMANIA_INFECTION
fold change
size
D
PTT+GC vs CTRL
KEGG_SPLICEOSOME
KEGG_ANTIGEN_PROCESSING_AND_PRESENTATION
KEGG_B_CELL_RECEPTOR_SIGNALING_PATHWAY
KEGG_LEISHMANIA_INFECTION
fold change
size
KEGG analysis (GSEA)
E
REACTOME_RRNA_PROCESSING
REACTOME_METABOLISM_OF_RNA
REACTOME_MRNA_SPLICING
REACTOME_AUTOPHAGY
REACTOME_CELLULAR_RESPONSE_TO_HEAT_STRESS
REACTOME_INTERFERON_ALPHA_BETA_SIGNALING
size
fold change
F
REACTOME_ATTENUATION_PHASE
REACTOME_HSF1_DEPENDENT_TRANSACTIVATION
REACTOME_CELLULAR_RESPONSE_TO_HEAT_STRESS
REACTOME_HSP90_CHAPERONE_CYCLE_FOR_STEROID_HORMONE_RECEPTORS_SHR_
REACTOME_INTERFERON_SIGNALING
REACTOME_INTERFERON_ALPHA_BETA_SIGNALING
size
fold change
Reactome analysis (GSEA)

Figure S2.

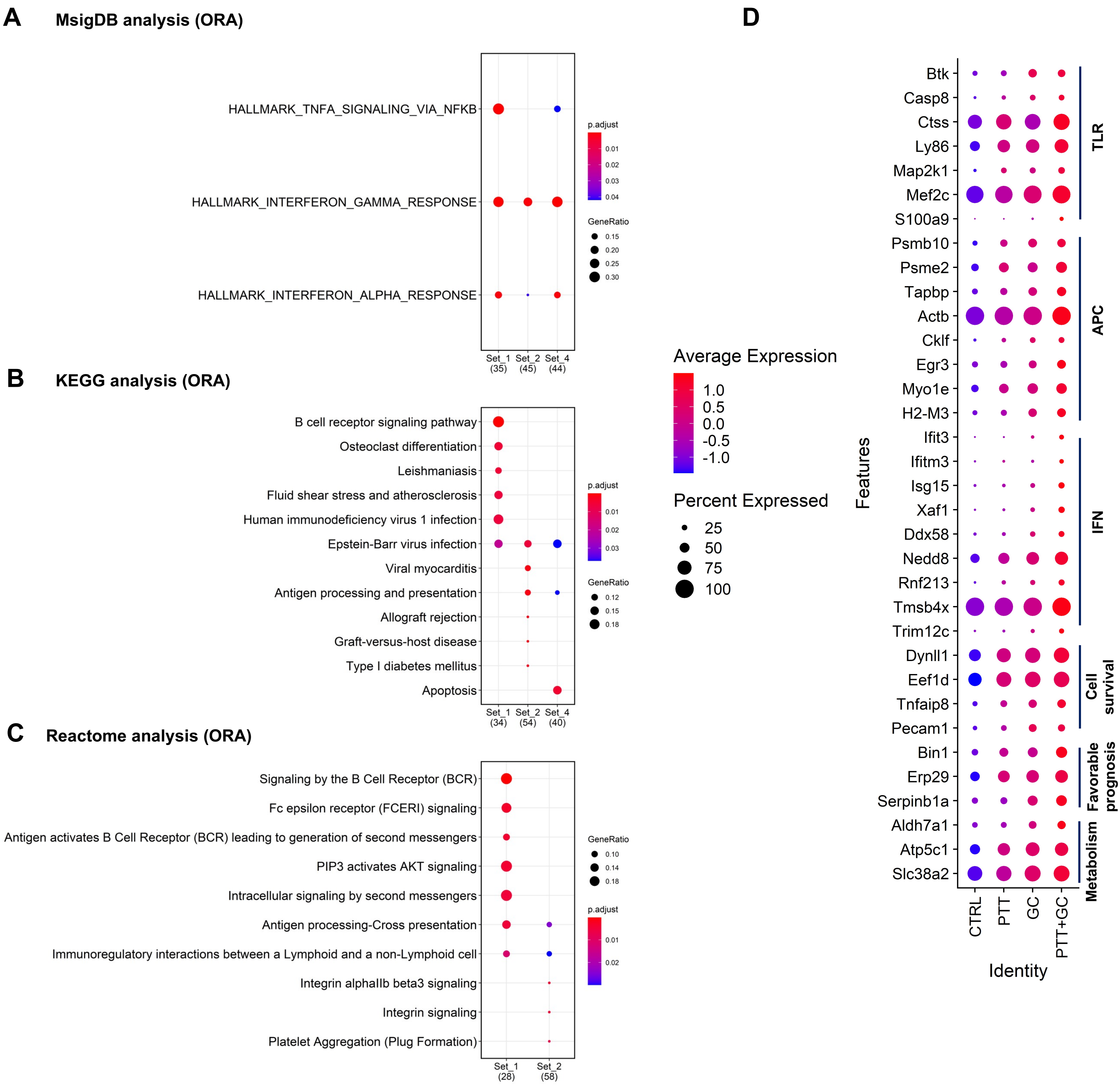

A MsigDB analysis (ORA)
HALLMARK_TNFA_SIGNALING_VIA_NFKB
HALLMARK_INTERFERON_GAMMA_RESPONSE
HALLMARK_INTERFERON_ALPHA_RESPONSE
Set_1 (35) Set_2 (45) Set_4 (44)
p.adjust
GeneRatio
B KEGG analysis (ORA)
B cell receptor signaling pathway
Osteoclast differentiation
Leishmaniasis
Fluid shear stress and atherosclerosis
Human immunodeficiency virus 1 infection
Epstein-Barr virus infection
Viral myocarditis
Antigen processing and presentation
Allograft rejection
Graft-versus-host disease
Type I diabetes mellitus
Apoptosis
Set_1 (34) Set_2 (54) Set_4 (40)
C Reactome analysis (ORA)
Signaling by the B Cell Receptor (BCR)
Fc epsilon receptor (FCERI) signaling
Antigen activates B Cell Receptor (BCR) leading to generation of second messengers
PIP3 activates AKT signaling
Intracellular signaling by second messengers
Antigen processing-Cross presentation
Immunoregulatory interactions between a Lymphoid and a non-Lymphoid cell
Integrin alphaIIb beta3 signaling
Integrin signaling
Platelet Aggregation (Plug Formation)
Set_1 (28) Set_2 (58)
D
Average Expression
1.0
0.5
0.0
-0.5
-1.0
Percent Expressed
25
50
75
100
Features
Btk
Casp8
Ctss
Ly86
Map2k1
Mef2c
S100a9
Psmb10
Psme2
Tapbp
Actb
Cklf
Egr3
Myo1e
H2-M3
Ifit3
Ifitm3
Isg15
Xaf1
Ddx58
Nedd8
Rnf213
Tmsb4x
Trim12c
Dynll1
Eef1d
Tnfaip8
Pecam1
Bin1
Erp29
Serpinb1a
Aldh7a1
Atp5c1
Slc38a2
TLR
APC
IFN
Cell survival
Favorable prognosis
Metabolism
CTRL
PTT
GC
PTT+GC
Identity

Figure S3.

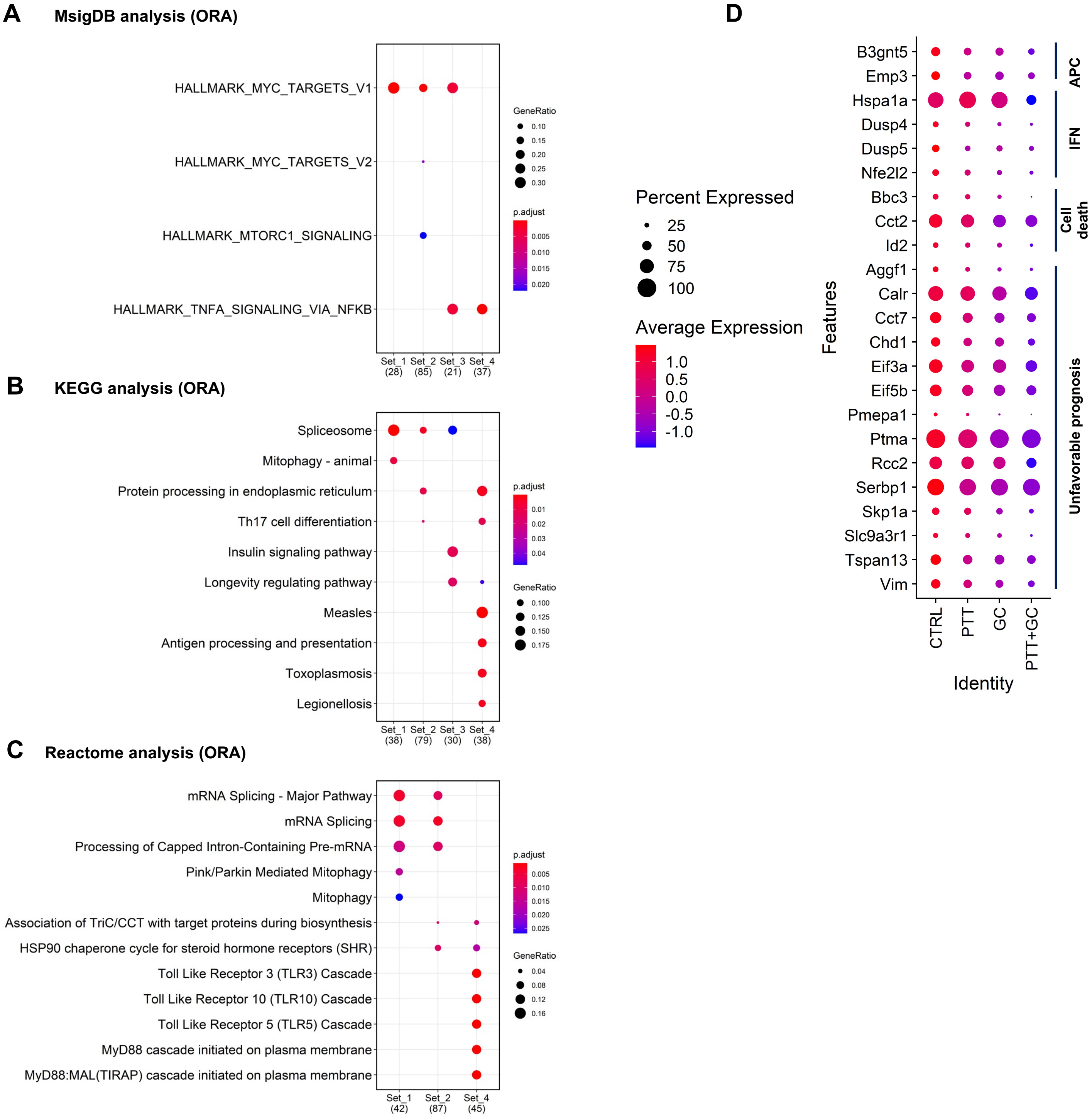
A
MsigDB analysis (ORA)
HALLMARK_MYC_TARGETS_V1
HALLMARK_MYC_TARGETS_V2
HALLMARK_MTORC1_SIGNALING
HALLMARK_TNFA_SIGNALING_VIA_NFKB
Set_1 (28)
Set_2 (85)
Set_3 (21)
Set_4 (37)
GeneRatio
0.10
0.15
0.20
0.25
0.30
p.adjust
0.005
0.010
0.015
0.020
B
KEGG analysis (ORA)
Spliceosome
Mitophagy - animal
Protein processing in endoplasmic reticulum
Th17 cell differentiation
Insulin signaling pathway
Longevity regulating pathway
Measles
Antigen processing and presentation
Toxoplasmosis
Legionellosis
Set_1 (38)
Set_2 (79)
Set_3 (30)
Set_4 (38)
p.adjust
0.01
0.02
0.03
0.04
GeneRatio
0.100
0.125
0.150
0.175
C
Reactome analysis (ORA)
mRNA Splicing - Major Pathway
mRNA Splicing
Processing of Capped Intron-Containing Pre-mRNA
Pink/Parkin Mediated Mitophagy
Mitophagy
Association of TriC/CCT with target proteins during biosynthesis
HSP90 chaperone cycle for steroid hormone receptors (SHR)
Toll Like Receptor 3 (TLR3) Cascade
Toll Like Receptor 10 (TLR10) Cascade
Toll Like Receptor 5 (TLR5) Cascade
MyD88 cascade initiated on plasma membrane
MyD88:MAL(TIRAP) cascade initiated on plasma membrane
Set_1 (42)
Set_2 (87)
Set_4 (45)
p.adjust
0.005
0.010
0.015
0.020
0.025
GeneRatio
0.04
0.08
0.12
0.16
D
Percent Expressed
25
50
75
100
Average Expression
1.0
0.5
0.0
-0.5
-1.0
Features
B3gnt5
Emp3
Hspa1a
Dusp4
Dusp5
Nfe2l2
Bbc3
Cct2
Id2
Aggf1
Calr
Cct7
Chd1
Eif3a
Eif5b
Pmepa1
Ptma
Rcc2
Serbp1
Skp1a
Slc9a3r1
Tspan13
Vim
APC
IFN
Cell death
Unfavorable prognosis
CTRL
PTT
GC
PTT+GC
Identity

Figure S4.

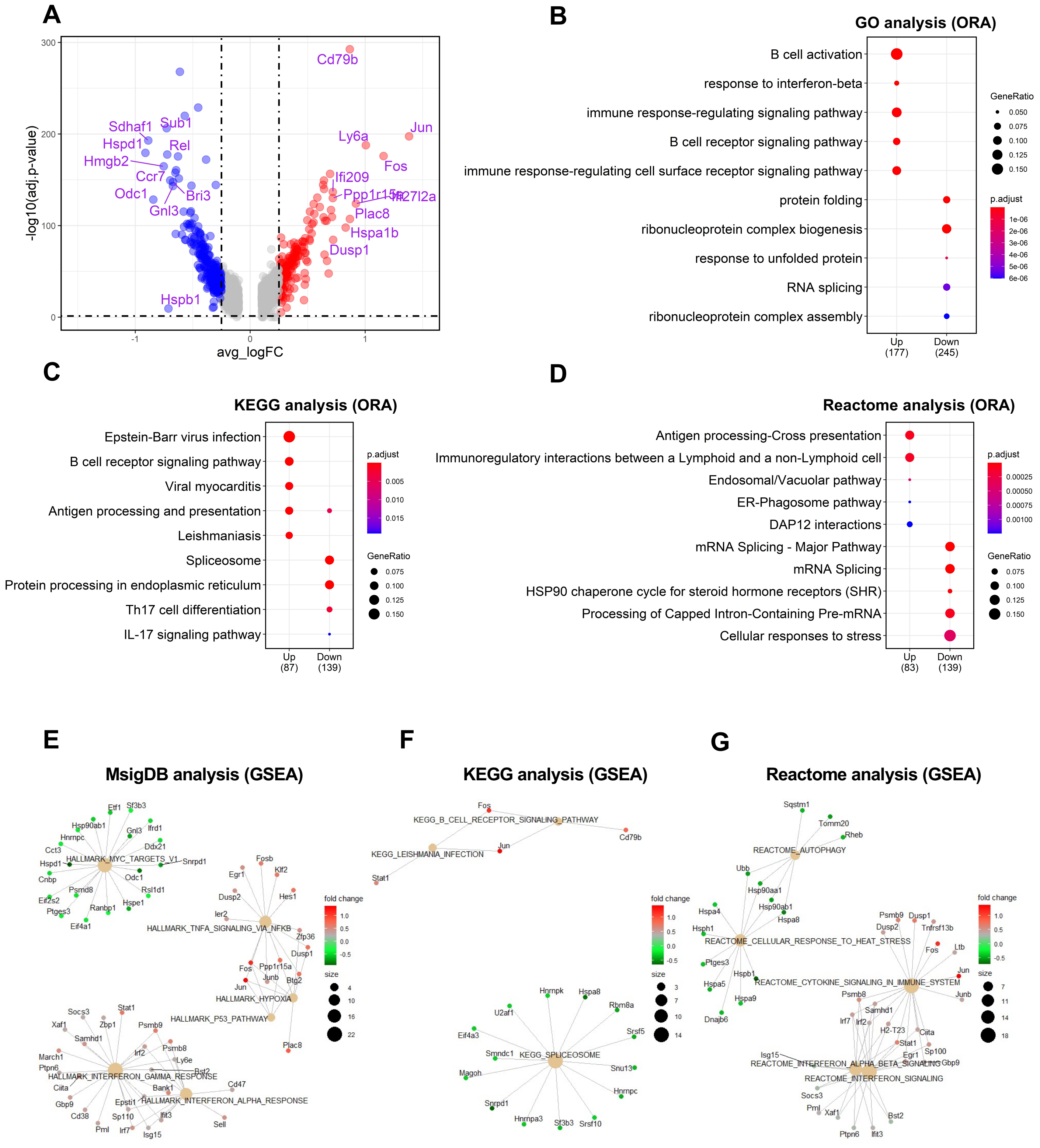
A
B
GO analysis (ORA)
C
KEGG analysis (ORA)
D
Reactome analysis (ORA)
E
MsigDB analysis (GSEA)
F
KEGG analysis (GSEA)
G
Reactome analysis (GSEA)
avg_logFC
-log10(adj.p-value)
B cell activation
response to interferon-beta
immune response-regulating signaling pathway
B cell receptor signaling pathway
immune response-regulating cell surface receptor signaling pathway
protein folding
ribonucleoprotein complex biogenesis
response to unfolded protein
RNA splicing
ribonucleoprotein complex assembly
Up (177)
Down (245)
Epstein-Barr virus infection
B cell receptor signaling pathway
Viral myocarditis
Antigen processing and presentation
Leishmaniasis
Spliceosome
Protein processing in endoplasmic reticulum
Th17 cell differentiation
IL-17 signaling pathway
Up (87)
Down (139)
Antigen processing-Cross presentation
Immunoregulatory interactions between a Lymphoid and a non-Lymphoid cell
Endosomal/Vacuolar pathway
ER-Phagosome pathway
DAP12 interactions
mRNA Splicing - Major Pathway
mRNA Splicing
HSP90 chaperone cycle for steroid hormone receptors (SHR)
Processing of Capped Intron-Containing Pre-mRNA
Cellular responses to stress
Up (83)
Down (139)
GeneRatio
p.adjust
fold change
size

Figure S5.

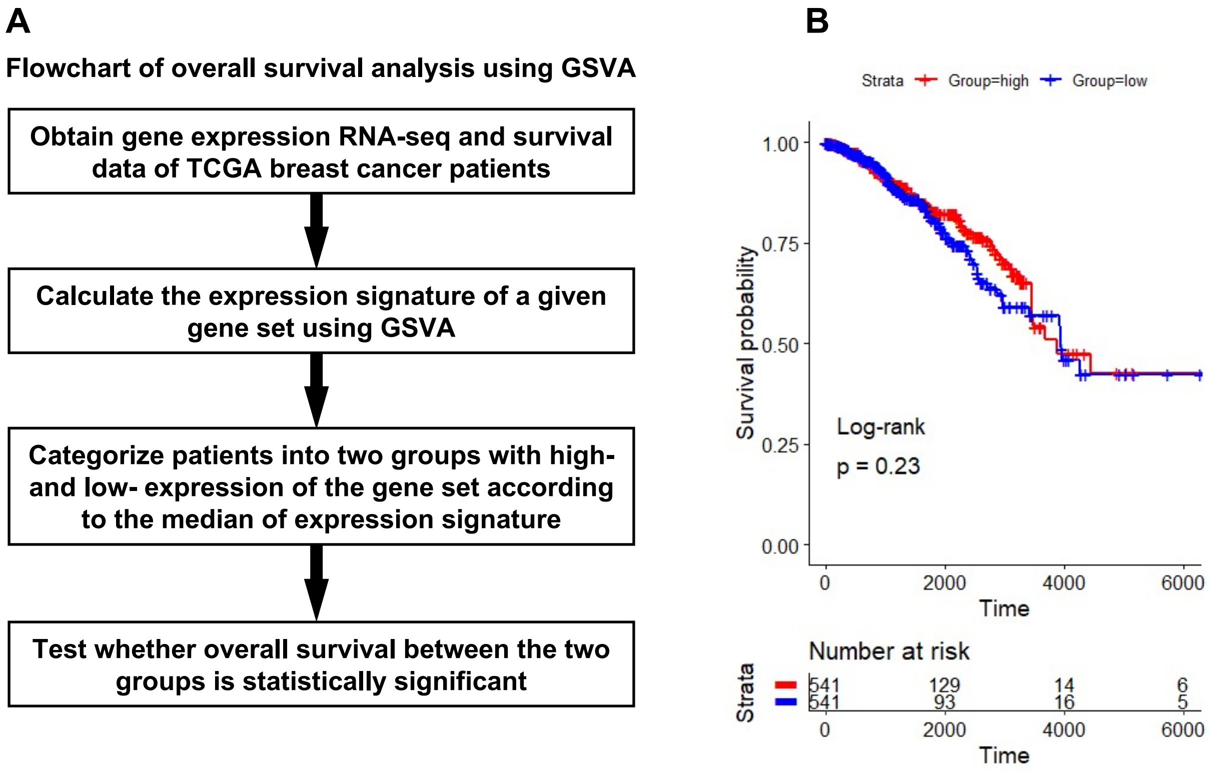
A
Flowchart of overall survival analysis using GSVA
Obtain gene expression RNA-seq and survival data of TCGA breast cancer patients
Calculate the expression signature of a given gene set using GSVA
Categorize patients into two groups with high- and low- expression of the gene set according to the median of expression signature
Test whether overall survival between the two groups is statistically significant
B
Strata
Group=high
Group=low
Survival probability
1.00
0.75
0.50
0.25
0.00
Log-rank
p = 0.23
0
2000
4000
6000
Time
Number at risk
Strata
541
129
14
6
541
93
16
5
0
2000
4000
6000
Time

Figure S6.

Function	Gene	Reference	Description
Toll-like receptors cascades	Btk	https://dx.doi.org/10.3389%2Ffimmu.2017.01986	Btk mediates pre-BCR or BCR downstream signals in B cells. It activates the MAPK cascade and realizes its biological functions by regulating the activity of transcription factors such as NF-KB.
	Casp8	https://dx.doi.org/10.1371%2Fjournal.ppat.1005910	Caspase-8 enzymatic activity regulates gene expression in response to bacterial infection as well as TLR signaling independently of apoptosis.
	Ctss	https://doi.org/10.1016/j.ccell.2020.03.016	CTSS regulates antigen processing and communication with CD4 + Tfh cells
	Ly86/MD-1	https://doi.org/10.1182/blood.v99.5.1699	Lymphocyte antigen 86 may cooperate with CD180 and TLR4 to mediate the innate immune response to bacterial lipopolysaccharide (LPS) and cytokine production.
	Mef2c	https://dx.doi.org/10.1038%2Fni.1609	We have identified Mef2c as a transcriptional effector of BCR signaling required for B cell activation and normal antibody responses.
	S100a9	https://doi.org/10.4049/jimmunol.1402301	Proinflammatory Proteins S100A8/S100A9 Activate NK Cells via Interaction with RAGE.
Antigen processing (-cross)/and presentation pathways	Psmb10	https://doi.org/10.1016/j.redox.2018.02.022	LMP10 is a positive regulator of NF-kB signaling.
	Psme2 (PA28)	https://doi.org/10.1016/s0161-5890(02)00099-8	PA28 selectively up-regulates the presentation of viral MHC class I epitopes.
	Tapbp (Tapasin)	https://doi.org/10.1002/immu.200390029	Tapasin is not only required for stabilization of TAP but also for optimization of the spectrum of bound peptides
Interferon signaling	Ifit3	https://doi.org/10.4049/jimmunol.1100963	Our study characterizes IFIT3 as an important modulator in innate immunity.
		https://doi.org/10.3389/fmolb.2019.00148	Interferon-induced protein with tetratricopeptide repeats (IFIT) genes are prominent interferon-stimulated genes (ISGs).
	Ifitm3	https://doi.org/10.1038/s41586-020-2884-6	IFITM3-dependent amplification of PI3K signalling, which in part acts downstream of the BCR, is critical for the rapid expansion of B cells with high affinity to antigen.
	Isg15	https://doi.org/10.18632/oncotarget.3372	We conclude that free ISG15 may have antitumor and immunoregulatory function in vivo.
	Xaf1	https://doi.org/10.1038/s41419-018-0867-4	XAF1 forms a positive feedback loop with IRF-1 to drive apoptotic stress response and suppress tumorigenesis.

	Ddx58 (RIG-1)	https://doi.org/10.1038/ncomms15138	RIG-I senses viral RNA and initiates an effective innate immune response for type I interferon production.
	Nedd8	https://doi.org/10.1186/s12974-019-1669-z	We present here the first evidence that the neuroinflammatory mediator IL-1beta facilitates ubiquitin ligase parkin/NEDD8 interactions.
	Rnf213	https://dx.doi.org/10.1038%2Fsrep13191	Our data illustrate that RNF213 plays unique roles in endothelial cells for proper gene expressions in response to inflammatory signals from environments.
	Tmsb4x	https://doi.org/10.1016/j.imbio.2011.04.002	Tbeta4 is regulated by IL-18 and is involved in IL-18-enhanced IFN-gamma secretion in NK cells.
	Trim12c	https://doi.org/10.4049/jimmunol.1402064	Trim12c stimulates type I IFN and NF-κB pathways.
Cell survival	Dynll1	https://dx.doi.org/10.1371%2Fjournal.pgen.1007010	Dynein light chain regulates adaptive and innate B cell development by distinctive genetic mechanisms.
	Eef1d	http://www.ncbi.nlm.nih.gov/pmc/articles/pmc6309335/	Eef1d is a partner for CD48, a component of co-signaling receptors expressed on the cell membrane of antigen presenting cells (APCs).
	Tnfaip8	https://doi.org/10.1002/mc.22740	TNFAIP8 regulates Hippo pathway through interacting with LATS1 to promote cell proliferation.
	Pecam1 (CD31)	https://doi.org/10.1182/blood-2002-01-0027	Platelet endothelial cell adhesion molecule-1 (PECAM-1/CD31) acts as a regulator of B-cell development, B-cell antigen receptor (BCR)–mediated activation, and autoimmune disease.
Favorable Prognosis	Bin1	https://doi.org/10.1038/onc.2017.217	BIN1 reverses PD-L1-mediated immune escape by inactivating the c-MYC and EGFR/MAPK signaling pathways in non-small cell lung cancer.
	Erp29	https://doi.org/10.1038/labinvest.2009.87	Overexpression of endoplasmic reticulum protein 29 regulates mesenchymal-epithelial transition and suppresses xenograft tumor growth of invasive breast cancer cells.
	Serpinb1a	https://doi.org/10.18632/oncotarget.6956	Data show that high serpin B1 protein (SERPINB1) gene expression was associated with favorable tumor response and prolonged survival under cisplatin-based chemotherapy.

Function	Gene	Reference	Description
repression of antigen-presenting	B3gnt5	https://dx.doi.org/10.1073%2Fpnas.0914298107	B3gnt5 KO B cells were more sensitive to the induction of intracellular phosphorylation signals on BCR stimulation and proliferated more vigorously than WT B cells.
		https://doi.org/10.1111/cns.13439	Patients with high B3GNT5 expression had a short overall survival.
	Emp3	https://dx.doi.org/10.1097%2FMD.0000000000009538	High EMP3 expression might be an independent indicator of unfavorable OS in GBM.
	Hspa1a	https://doi.org/10.1007/978-94-007-5943-5_5	Intracellular HSP70 protects the cell and restricts cytokine production
repression of IFN	Dusp4	https://dx.doi.org/10.1073%2Fpnas.1109797109	Increased DUSP4 expression in activated T cells in the elderly in part accounts for defective adaptive immune responses.
	Dusp5	https://dx.doi.org/10.3390%2Fijms20112710	Dusp4 and Dusp5 inhibits MAPK and therefore restrict cytokine expression.
	Nfe2l2(Nrf2)	https://dx.doi.org/10.1165%2Frcmb.2010-0321OC	Nrf2 knockout enhances the inflammation and T lymphocyte function.
regulation of cell fate	Bbc3 (PUMA)	https://doi.org/10.1182/blood-2011-04-347096	Puma is a major regulator of memory B lymphocyte survival and therefore a key molecule in the control of the immune response.
		https://doi.org/10.1007/s12094-013-1010-8	High expression of PUMA is associated with lymph node metastasis and invasion in gallbladder adenocarcinoma.
	Id2	https://doi.org/10.1073/pnas.0802550106	Inhibition of id2 is required for B cell lineage.
unfavorable prognosis	Aggf1	https://doi.org/10.12659/msm.903248	High expression of AGGF1 predicts poor prognosis in gastric cancer patients.
	Calr	https://doi.org/10.1155/2019/8792640	Plasma calreticulin level was positively correlated with the severity of sepsis and predicted patient mortality.
	Cct2	https://doi.org/10.1038/s41598-019-43556-1	Overexpression of other four HSPs – HSP90AA1, CCT1, CCT2, CCT6A resulted in unfavorable prognosis for breast cancer patients.
		https://doi.org/10.1186/1477-7819-11-143	positive expression of CCT2 and PDIA3 was negatively correlated with poor postoperative patient survival and positively correlated with high mortality.
	Chd1	https://doi.org/10.1007/s10620-017-4641-8	Increased CHD1L protein expression was significantly associated with poor overall survival.

Eif5b	https://doi.org/10.1038/s43018-020-0056-0	eIF5B overexpression, which is frequent in lung adenocarcinomas and associated with poor prognosis, is sufficient to induce PD-L1.
Pmepa1(TMEPAI)	https://doi.org/10.1111/cas.12355	TMEPAI is constitutively and highly expressed in many types of cancer and is associated with poor prognosis.
Rcc2	https://doi.org/10.1158/1078-0432.ccr-16-2909	Patients with LUAD with higher expression of RCC2 had shorter overall survival.
Serbp1	https://doi.org/10.1186/s13059-020-02115-y	High SERBP1 expression is prevalent in GBMs and correlates with poor patient survival and poor response to chemo- and radiotherapy
Skp1a	https://doi.org/10.1158/1078-0432.ccr-18-3631	IHC analysis revealed that cytoplasmic expression of SKP1 was significantly associated with SFN positivity, tumor malignancy, and poorer patient outcome.
	https://doi.org/10.18632/oncotarget.5547	Skp1 was overexpressed in 36/64 (56.3%) of non-small cell lung cancers, and elevated Skp1 was associated with poor prognosis.
Slc9a3r1 (NHE-RF1/EBP50)	https://doi.org/10.18632/oncotarget.8751	NHERF1 was upregulated in high grades compared with low grades. Increased NHERF1 expression was correlated with poor prognosis and poor survival.
Vim (Vimentin)	https://doi.org/10.1245/s10434-019-07891-x	Higher vimentin expression (p = 0.018) was associated with significantly shorter overall survival in PDAC patients.
